# Hydrogel‐Based Vat Photopolymerization of Ceramics and Metals with Low Shrinkages via Repeated Infusion Precipitation

**DOI:** 10.1002/adma.202504951

**Published:** 2025-09-24

**Authors:** Yiming Ji, Ying Hong, Dhruv R. Bhandari, Daryl W. Yee

**Affiliations:** ^1^ Institute of Electrical and Micro Engineering École Polytechnique Fédérale de Lausanne (EPFL) Rue de la Maladière 71b Neuchâtel 2000 Switzerland; ^2^ Department of Metallurgical Engineering & Materials Science Indian Institute of Technology Bombay Main Gate Rd, IIT Area, Powai Mumbai Maharashtra 400076 India

**Keywords:** additive manufacturing, in situ synthesis, polymer‐derived materials, vat photopolymerization

## Abstract

Vat photopolymerization (VP) is a powerful tool for the fabrication of architected ceramic and metal structures. However, conventional methods of ceramic/metal VP, such as with the use of slurries or organic–inorganic hybrid resins, have challenges with viscosities, light‐scattering, and limited material compositions. Recently, the use of metal‐salt solutions has emerged as a promising approach for the VP of ceramics and metals. While versatile and accessible, the process is accompanied by a significant amount of shrinkage, which causes warping, porosity, and structural damage. Here, a versatile method is presented for fabricating dense architected ceramics and metals with low conversion linear shrinkages. Central to this method is a post‐fabrication repeated infusion‐coprecipitation process that progressively increases the metal loading in the 3D “blank” hydrogels. Thermal treatment of these high metal content hydrogels then converts them into ceramic or metal architectures. To demonstrate the versatility of this approach, a variety of 3D ceramic and metal structures with shrinkages as low as 20% while maintaining densities >80% is fabricated. This infusion‐precipitation‐based process thus enables the VP of high‐quality ceramics and metals, which is necessary for the fabrication of advanced architected materials and devices.

## Graphical abstract

This work introduces an in situ precipitation strategy for the hydrogel‐based vat photopolymerization of architected ceramics and metals with reduced shrinkages and improved densities. Repeated infusion‐precipitation significantly increases metal loading within the hydrogels, which improves part quality after thermal conversion. This strategy offers a versatile platform for the fabrication of advanced structural and functional materials and next‐generation 3D‐architected devices.

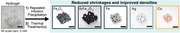

## 1 Introduction

Architected materials are an emerging class of materials that leverage 3D structural geometry to access functionalities and properties inaccessible through composition and/or microstructure optimization alone.^[^ ^1^, ^2^ ^]^ Over the past decades, advances in our understanding of architecture‐property relationships and our manufacturing tools have led to the development of 3D nano‐ and micro‐architected materials with enhanced or emergent properties—from extreme mechanical behaviors^[^ ^3^, ^4^, ^5^ ^]^ to exotic optical properties^[^ ^6^, ^7^, ^8^ ^]^—that cannot be achieved with conventionally processed materials. Architected materials thus have the potential to address society's increasing demand for high‐performance devices and enable next‐generation technologies in energy,^[^ ^9^, ^10^, ^11^ ^]^ biomedical,^[^ ^12^, ^13^, ^14^ ^]^ and sensing^[^ ^15^, ^16^, ^17^ ^]^ applications.

Today, architected materials are predominantly fabricated with additive manufacturing (AM) technologies since they are able to produce 3D structures with arbitrary complexity over a wide range of length scales.^[^ ^18^ ^]^ In particular, vat photopolymerization (VP)—a category of AM processes that utilizes spatial photopolymerization of a liquid resin in a vat to fabricate 3D structures^[^ ^19^ ^]^—is often employed due to its ability to achieve small feature sizes^[^ ^20^ ^]^ and rapid fabrication speeds.^[^ ^21^ ^]^ However, a key limitation of VP is that because it is fundamentally a photopolymerization process, it is challenging to fabricate non‐polymeric materials with it. Since polymers offer limited structural and functional properties, this material constraint restricts the possible range of functionalities and use‐cases of devices fabricated with VP. Consequently, significant efforts have been made to develop methods that enable the VP of non‐photopolymerizable materials such as metals and ceramics.^[^ ^22^, ^23^ ^]^

The most established methods for the VP of metals and ceramics currently revolve around the use of either a photosensitive slurry or an inorganic‐organic hybrid photoresin. With the slurry method, ceramic/metal powders are dispersed in a liquid photoresin and then used with a VP technique to form a composite polymer structure. The composite “green body” is then thermally treated in the appropriate atmosphere(s) to obtain the desired metal or ceramic structure.^[^ ^24^, ^25^ ^]^ Although highly accessible and versatile,^[^ ^26^, ^27^, ^28^, ^29^ ^]^ the inclusion of particles in the resin increases its viscosity^[^ ^30^ ^]^ and causes light‐scattering,^[^ ^31^, ^32^, ^33^ ^]^ which negatively impacts the quality and feature sizes of the resulting ceramic and metal structures. An alternative approach that circumvents these issues is to use particle‐free inorganic‐organic hybrid photoresins instead. These hybrid photoresins consist of photopolymerizable (macro)monomers that contain metal heteroatoms, forming polymers that act as both the binder and the inorganic precursor. Similar to the slurry method, the printed inorganic‐organic hybrid polymers are thermally treated to yield the desired ceramic or metal structures.^[^ ^34^, ^35^, ^36^, ^37^ ^]^ Since these photoresins are particle‐free, they have low viscosities and have little issues with light‐scattering, allowing them to fully reach the small features sizes afforded by VP.^[^ ^38^ ^]^ However, the small selection of inorganic–organic (macro)monomers available limits the compositions of the ceramics and metals that can be fabricated with this approach. In addition, the shrinkage associated with the polymer‐to‐ceramic conversion process can be significant, resulting in damage to the final structure.

In recent years, the use of aqueous metal salt solutions has emerged as a promising alternative strategy for the VP of ceramics and metals. In brief, polymers that contain aqueous metal salt solutions are first fabricated and then thermally treated to convert them into ceramics and metals.^[^ ^39^, ^40^, ^41^, ^42^ ^]^ Compared to the slurry and hybrid resin approaches, the metal salt method offers several key advantages: a) a wide variety of metal salts are commercially available; b) multicomponent inorganic materials can be made by simply dissolving the constituent metal salts in the appropriate ratios;^[^ ^43^, ^44^, ^45^, ^46^ ^]^ and c) the particle‐free aqueous resins do not scatter light^[^ ^47^, ^48^, ^49^ ^]^ and have low viscosities. The metal salt method thus combines the simplicity and versatility of the slurry method with the small feature sizes afforded by the hybrid resin method. Building on this, we recently developed a process called hydrogel infusion additive manufacturing (HIAM), where “blank” hydrogels are infused with a variety of metal salt solutions post‐fabrication and then thermally treated to convert them into metal oxides or metals.^[^ ^50^ ^]^ HIAM is thus unique in that it enables a single resin composition to be used for the fabrication of an almost infinite array of non‐polymeric materials. More importantly, it highlights a new paradigm of AM where material selection occurs after, rather than before, structure fabrication. However, despite its accessibility and versatility, the utility of HIAM, and the metal salt approach in general, has been limited by the significant shrinkages (50–90%) that occur during the polymer‐to‐ceramic conversion process.^[^ ^50^, ^51^, ^52^, ^53^, ^54^, ^55^ ^]^ This excessive shrinkage causes significant warping, cracking, and porosity in the final ceramic/metal parts, often rendering them too fragile for reliable use in practical applications.

Since shrinkage is inversely proportionate to the amount of metal precursors in the hydrogel, recent research in this area has largely focused on the development of strategies that enhance metal‐ion loading in the hydrogels. Wang and colleagues developed charged ion‐exchangeable hydrogels that could be infused with up to 20 wt% of Cu^2+^ ions, which was twice the previous limit.^[^ ^56^ ^]^ Their copper complexed polymers could be thermally converted into copper structures with an associated linear shrinkage of approximately 60%. Xiong and colleagues showed that 1‐vinylimidazole and acrylic acid synergistically coordinated with metal ions in water and used them to increase metal‐ion loading significantly. Although not strictly an infusion‐based process, metal oxides structures fabricated using their approach exhibited polymer‐to‐ceramic linear shrinkages between 30% and 55%.^[^ ^57^ ^]^ Although these strategies have been effective in reducing some of the conversion shrinkage, their efficacies are fundamentally limited as the amount of metal‐ions in the hydrogel is constrained by the solubility limit of the metal salts in water or the binding capacity of the hydrogel. In addition, since a large volume fraction of the ion‐infused hydrogels is water, significant shrinkage will necessarily occur during drying.

In this study, we describe an infusion‐precipitation strategy to fabricate 3D ceramic and metal structures with low conversion shrinkages and improved densities. Using our approach, we chemically transform 3D “blank” hydrogels into hydrogel composites with high metal contents. Subsequent thermal treatment of these composites converts them into ceramics and metals with low linear shrinkages and high densities. To demonstrate the versatility of our approach, we fabricated a variety of metal oxides and metals, including Fe_2_O_3_, SrFe_12_O_19_, Fe, Cu, and Ag. Importantly, our method allows us to obtain equivalent metal ion loadings within the polymer templates of up to 79 wt%, which significantly exceeds the state of the art. Such high metal loadings drastically reduce shrinkage (as low as 20% for some compositions) and warping after thermal conversion, while simultaneously increasing material density. Consequently, structures created using our approach boast significantly improved mechanical properties. Taken together, these findings demonstrate the low shrinkage VP of dense architected ceramics and metals, which could pave the way towards their use in practical applications.

## 2 Results and Discussion

### 2.1 Increasing Metal Loading via Repeated Infusion‐Coprecipitation Cycles

**Figure 1:**
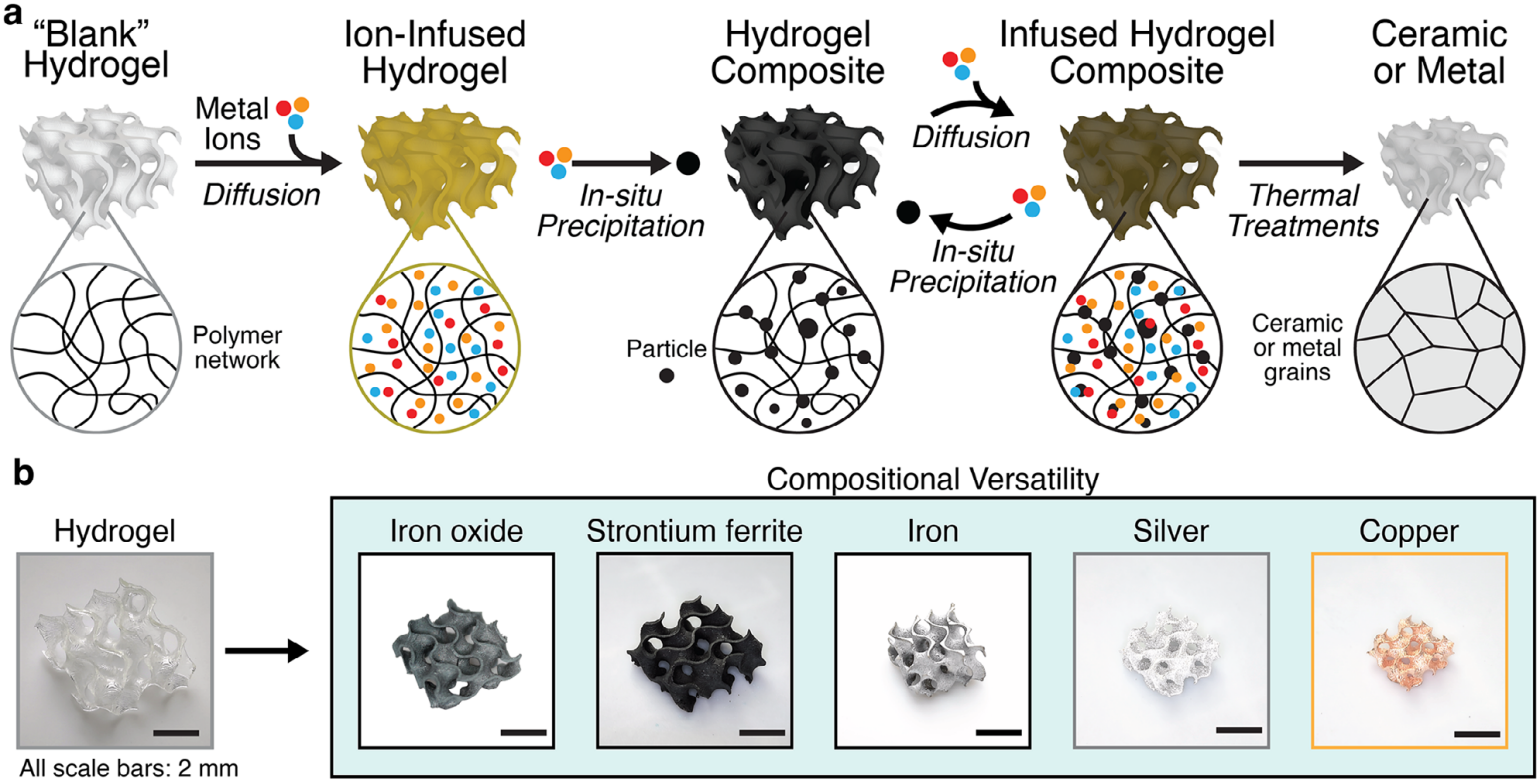
a) Schematic of the hydrogel infusion and precipitation process for the VP of ceramics and metals. b) Optical images of metal oxides and metals fabricated via our infusion‐precipitation based VP process.

The amount of metal‐ions that can be loaded into a hydrogel is limited by its equilibrium adsorption capacity. Since this limit is specific to metal‐ions, a facile strategy to increase overall metal loading is to incorporate non‐ion‐based metal precursors as well. Inspired by prior work on biomineralized hydrogels,^[^ ^58^, ^59^ ^]^ we hypothesized that we could achieve this by periodically precipitating the infused metal‐ions in situ into metal‐containing particles. Since the precipitation process depletes the hydrogel of metal‐ions, the infusion‐precipitation cycles can be repeated to progressively increase the overall metal content in the hydrogel^[^ ^60^, ^61^ ^]^ (**Figure** **1**a). Our approach thus decouples metal loading from hydrogel ion‐adsorption capacity since ionic and non‐ionic metal precursors are now used. Importantly, since the metal‐containing particles are only formed in situ post‐fabrication, our infusion‐precipitation process retains all the hallmarks of metal‐salt solution approaches: i) scatter‐free fabrication, ii) compositional versatility, iii) the ability for a single “blank” polymer to be transformed into a multitude of other materials (Figure 1b). The high metal‐content hydrogel composites are then thermally converted into ceramics or metals with significantly reduced shrinkages (Figure 1a). To demonstrate the compositional versatility of our approach, we fabricated 3D gyroid structures of iron oxide, strontium hexaferrite, iron, copper, and silver (Figure 1b).

The “blank” hydrogel resin was first prepared by mixing poly(ethylene glycol) diacrylate (PEGda, *M*_n_ = 700 g mol^−1^), water, lithium phenyl‐2,4,6‐trimethylbenzoylphosphinate, and tartrazine together (Table S1, Supporting Information). The hydrogel resin was then used with a digital light processing (DLP) printer to fabricate gyroid lattices with wall thicknesses of 150 µm (Figure S1, Supporting Information). Post‐fabrication, the gyroid lattices were developed in water overnight to remove any unreacted resin and swell it to its equilibrium size. The general infusion‐precipitation process is then as follows: the “blank” gyroids are immersed in a metal salt solution to infuse them with metal ions. However, instead of directly thermally converting the ion‐infused hydrogels into metal oxides, we form an intermediate hydrogel composite by using a precipitating agent to induce the in situ formation of metal‐containing nanoparticles. The infusion‐precipitation cycle is then repeated multiple times to increase the mass of metal‐containing nanoparticles in the hydrogel composite. Once the desired mass fraction of nanoparticles in the composite is reached, they are thermally treated in the appropriate atmospheres to obtain either metal oxide or metal structures.

### 2.2 In Situ Synthesis of Nanoparticles

As the goal of our approach is to maximize metal loading in the hydrogel templates, we sought to understand how to efficiently increase the mass of nanoparticles precipitated within the hydrogels. For ease of discussion, we will use the ammonia‐induced in situ coprecipitation of iron oxide nanoparticles^[^ ^60^ ^]^ as an example to illustrate the key factors that impact mass loading. As expected, we found that increasing the concentration of iron‐ions in the infusion solution led to an almost proportional increase in the mass of the hydrogel after infusion (Figure S2a, Supporting Information). Based on these results, the 1.5 m FeCl_2_ and 2.7 m FeCl_3_ solution was used for infusion since it resulted in the largest mass increase after (≈80%). Increasing the concentration beyond this was not possible due to solubility issues.

We next determined the impact of temperature on the infusion process (Figure S2b, Supporting Information). Unsurprisingly, increasing the infusion temperature decreased the time needed to reach the equilibrium adsorption capacity—at 80 °C, equilibrium was reached after 30 min, whereas it took 60 min at 65 °C. However, as metal ions can form acidic solutions, and the rate of acid‐catalyzed hydrolysis of acrylic esters is strongly dependent on temperature, we posited that repeated infusion at 80 °C would result in significant degradation to the hydrogel matrix. Indeed, we observed a slight decrease in the mass change after 180 minutes at 80 °C, which suggested that some hydrogel degradation had occurred. As such, to balance infusion efficiency with hydrogel stability, ion‐infusion was conducted at 65 °C for 60 min.

**Figure 2:**
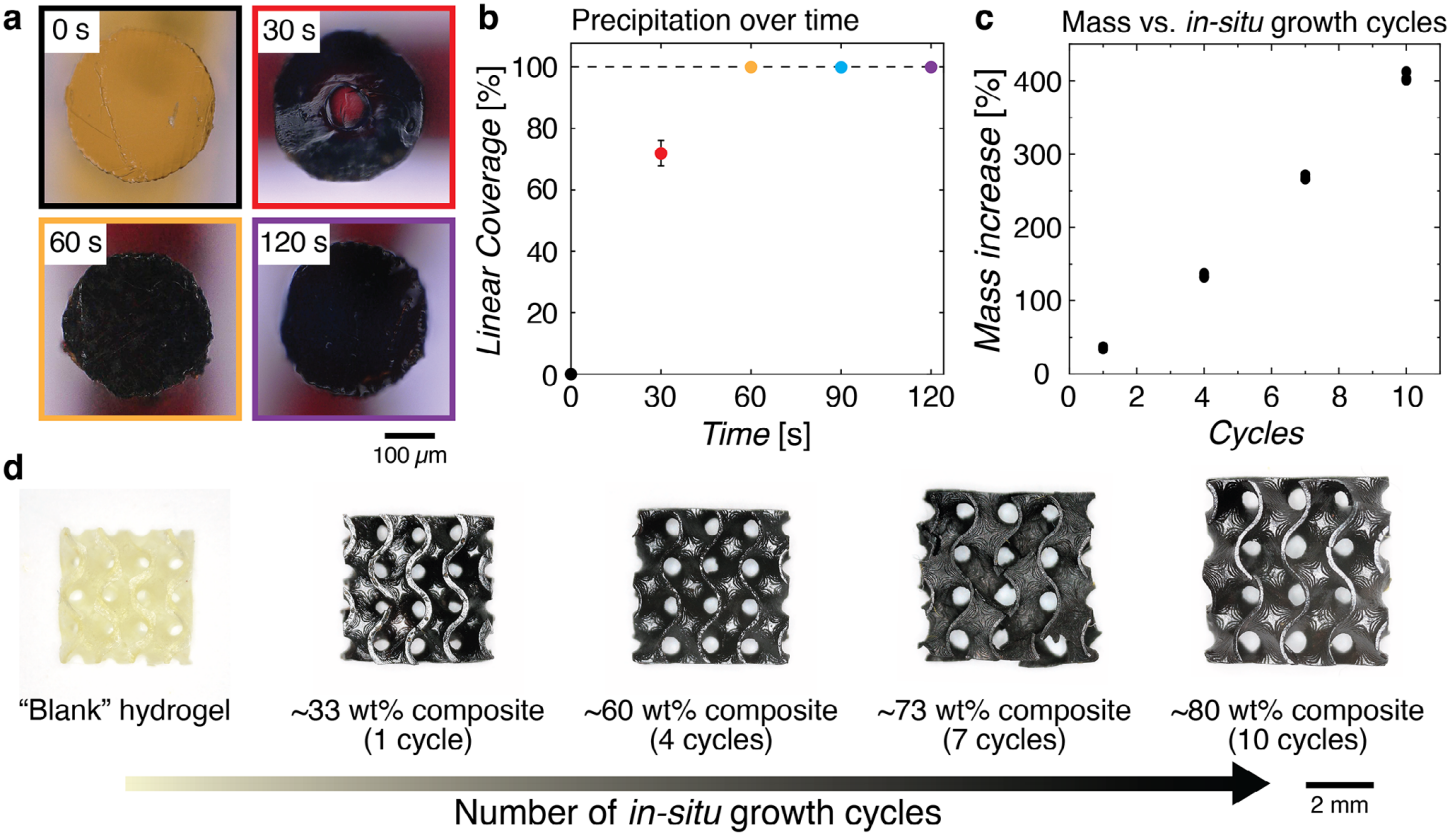
a) Cross‐sections of iron‐infused hydrogel pillars after different exposure times to ammonium hydroxide. b) Extent of in situ coprecipitation as a function of ammonium hydroxide exposure time. Linear coverage is measured by the length of the growth front as compared to the radius of the pillar. c) Mass increase of the hydrogel as a function of the number of infusion‐coprecipitation cycles. d) Optical image of the dried “blank” hydrogel and IONP composites. The size of the composites increased with the number of in situ growth cycles.

The iron‐ion infused hydrogels were then immersed in a 30% ammonium hydroxide solution to initiate the in situ coprecipitation of iron oxide. To obtain some insight into the in situ coprecipitation process, we examined cross‐sections of infused hydrogel pillars (350 µm diameter) at varying exposure times to ammonium hydroxide at room temperature (**Figure** **2**a). Consistent with the diffusion of ammonium hydroxide from solution into the hydrogel, we observed the formation of a black ring that rapidly advanced inward from the surface of the pillar towards its core over time. The entire cross‐section of the pillar turned black after 60 seconds, with no further visual changes beyond that, which suggests that the coprecipitation reaction was complete by that time (Figure 2b). Considering that the 3D gyroids had a wall thickness (150 µm) smaller than that of the pillars, we expect the coprecipitation reaction to be complete within 60 seconds. After coprecipitation, the dried masses of the gyroid hydrogel composites were approximately 25% higher than that of their initial “blank” dried masses. To ensure that the iron‐containing particles were incorporated within the hydrogels and not just deposited on their surfaces, we measured the masses of the hydrogel composites before and after 30 s of ultrasonication in water. No significant mass changes were observed, which indicated that the precipitates were bound within the hydrogel.

Post‐coprecipitation, the hydrogel composites were completely depleted of iron ions, which allowed us to repeat the infusion‐coprecipitation process described above. We found that the mass of the hydrogel composite could be increased significantly, with an almost linear relationship between the mass change and the number of infusion‐coprecipitation cycles (Figure 2c). After 10 infusion‐coprecipitation cycles, the mass of the dried hydrogel composite was 4 times that of the dried initial “blank hydrogel,” i.e., our composites contained up to 80 wt% of iron‐containing particles. Assuming that the mass change was solely attributed to the in situ generation of Fe_3_O_4_ particles, this meant that our hydrogels contained an equivalent iron‐ion loading of 58 wt%, which is significantly higher than the state of the art (Discussion S1, Supporting Information). It is important to emphasize that the VP of composites with such high mass fractions of light‐absorbing particles is challenging, even with established slurry printing methods.^[^ ^62^ ^]^ For example, the fabrication of iron‐based composites, as we have shown here, is difficult since iron and iron oxide particles strongly absorb light,^[^ ^63^ ^]^ agglomerate due to their magnetic properties,^[^ ^64^ ^]^ and can cause detrimental autocatalysis of the polymerization reaction.^[^ ^65^ ^]^ To date, the highest reported 3D printable iron‐slurry is approximately 30 wt%.^[^ ^63^ ^]^ Our ability to fabricate composites with significantly higher loadings highlights the utility of our in situ precipitation strategy. The increase in mass of the composite was also accompanied by an increase in volume of the composite (Figure 2d), with an almost linear relationship between the number of growth cycles and the linear expansion of the structure (Figure S3, Supporting Information). This was expected as the polymer network had to expand to accommodate the growth of the particles within the free volume of the polymer. We observed that 10 cycles of iron oxide growth was the limit with our gyroid structures—beyond that, the hydrogel composites were fragile, difficult to remove from the ammonium hydroxide solution, and often exhibited significant cracking upon drying (vide infra).

Energy‐dispersive X‐ray spectroscopy (EDS) line scans taken from cross‐sections of the composite structures showed that iron was detected across the entire cross‐section (Figure S4, Supporting Information), which corroborated the optical images in Figure 2a. However, the size of the particles, as determined via scanning electron microscopy (SEM), was observed to differ across the cross‐section of the structure. The 10‐cycle iron oxide composites had particle diameters of 22 ± 6 and 27 ± 8 nm at the core and surface of the structure respectively. Despite the size differences, the density of particles across the cross‐sections appeared to be qualitatively similar (Figure S5, Supporting Information). We posit that the smaller particle sizes at the center of the composite is likely due to a lower concentration of iron‐ions present during coprecipitation. As shown in Figure 2a, the center of the pillar was the last region to undergo the coprecipitation reaction. This delay allowed the iron ions initially present in the core to diffuse outward toward the surface, effectively reducing their concentration in the center during the coprecipitation reaction. The size and spatial distribution of the iron‐containing particles in the 7‐cycle composites are shown in Figure S5 (Supporting Information). The size of the particles in the 4‐cycles and 1‐cycle composites could not be accurately determined due to their small sizes (<20 nm), the large volume fraction of insulating polymer, and the general difficulty of imaging magnetic particles with electron microscopy.^[^ ^66^ ^]^

Although we only discussed the infusion and in situ coprecipitation of iron oxide, the factors that impact their growth are expected to be similar for other in situ nanoparticle syntheses, namely: concentration of metal‐ion solution, infusion time and temperature, concentration of precipitating reagent, and precipitation time and temperature. It is important to note that optimizing the infusion conditions to maximize the mass of particle growth per cycle is not always necessary as, even with sub‐optimal growth, the infusion‐precipitation process can just be repeated until the desired particle weight fraction in the composite is achieved.

As alluded to earlier, hydrogel composites with different nanoparticle compositions can be made by changing the reagents used during the infusion‐precipitation process. To demonstrate this compositional versatility, we fabricated copper and silver hydrogel composites via the direct reduction of their respective metal‐ion infused hydrogels using sodium borohydride (Figure S6, Supporting Information). Similar to the in situ precipitation of iron oxide, we observed that metal formation (Cu or Ag) was initiated at the surface and advanced towards the core of the material, reaching completion within 60 seconds (Figure S7, Supporting Information). At the infusion and precipitation conditions employed (See Experimental section), the maximum number of growth cycles that could be sustained for the Cu and Ag gyroid composites were 7 and 5 respectively, which resulted in metal particle loadings of ≈68 wt% and ≈79 wt% respectively (Figures S8 and S9, Supporting Information). Such high loadings of metals could be achieved with a lower number of growth cycles because higher concentrations of metal salt solutions could be used during infusion. Similar to the iron oxide system, increasing the number of growth cycles further resulted in cracking and composite failure. We hypothesize that the reduced number of growth cycles achievable with the sodium borohydride systems were primarily due to the generation of H_2_ gas during the metal‐ion reduction process; for reference, no gases were generated during the ammonia‐induced precipitation of iron oxide. As more metal particles grew within the free volume of the hydrogel, the generated H_2_ gases likely encountered a more tortuous path to reach the surface and escape. Thus, at a sufficiently high wt% of particles, gas buildup created internal pressure, which then lead to crack formation. However, despite the reduced number of growth cycles, the metal loadings of the Cu and Ag composites were actually higher than that of the iron oxide composites since metal particles were formed directly during the reduction process, instead of metal oxides that have a lower wt% of metal. Taken together, these results demonstrate that our infusion–precipitation approach can be generalized to multiple nanoparticle systems, enabling the fabrication of diverse hydrogel composites. Importantly, we show that high metal loadings can be achieved in all our systems, which is critical for subsequent ceramic or metal conversion.

### 2.3 Thermal Conversion of the Polymer Composites into Metals

The hydrogel composites can then be thermally treated to convert them into 3D ceramic or metal structures. To further increase metal loading, an additional infusion step prior to thermal treatment can be conducted. However, we observed that the effectiveness of this step depended strongly on the type of metal salt used. Infusion of metal nitrates solutions always led to extensive cracking after thermal treatment, likely due to the strong oxidizing nature of nitrates, which facilitates rapid gas evolution^[^ ^67^ ^]^ (see Discussion S2, Supporting Information for more details). Importantly, we observed that the drying process prior to thermal treatment played a critical role in the structural integrity of the composite, and by extension, the quality of the resulting ceramic or metal structure. For example, although the hydrated infused 10‐cycle iron oxide hydrogel composites had no observable internal cracks (Figure S10a, Supporting Information), rapid drying of the composite resulted in the formation of cracks both within and on the surface of the structure (Figure S10b, Supporting Information), which remained in the iron oxide structure after calcination (Figure S11, Supporting Information). Slow drying significantly reduced defect formation, with the best results observed when slow drying at room temperature in the presence of hygroscopic silica gel (Figure S10c, Supporting Information). It is important to note that due to the small feature sizes of the structures, the stress on the network induced by the in situ growth of particles, and the loss of polymer ductility due to the increasing amount of fillers, the composites have to be handled carefully.

**Figure 3:**
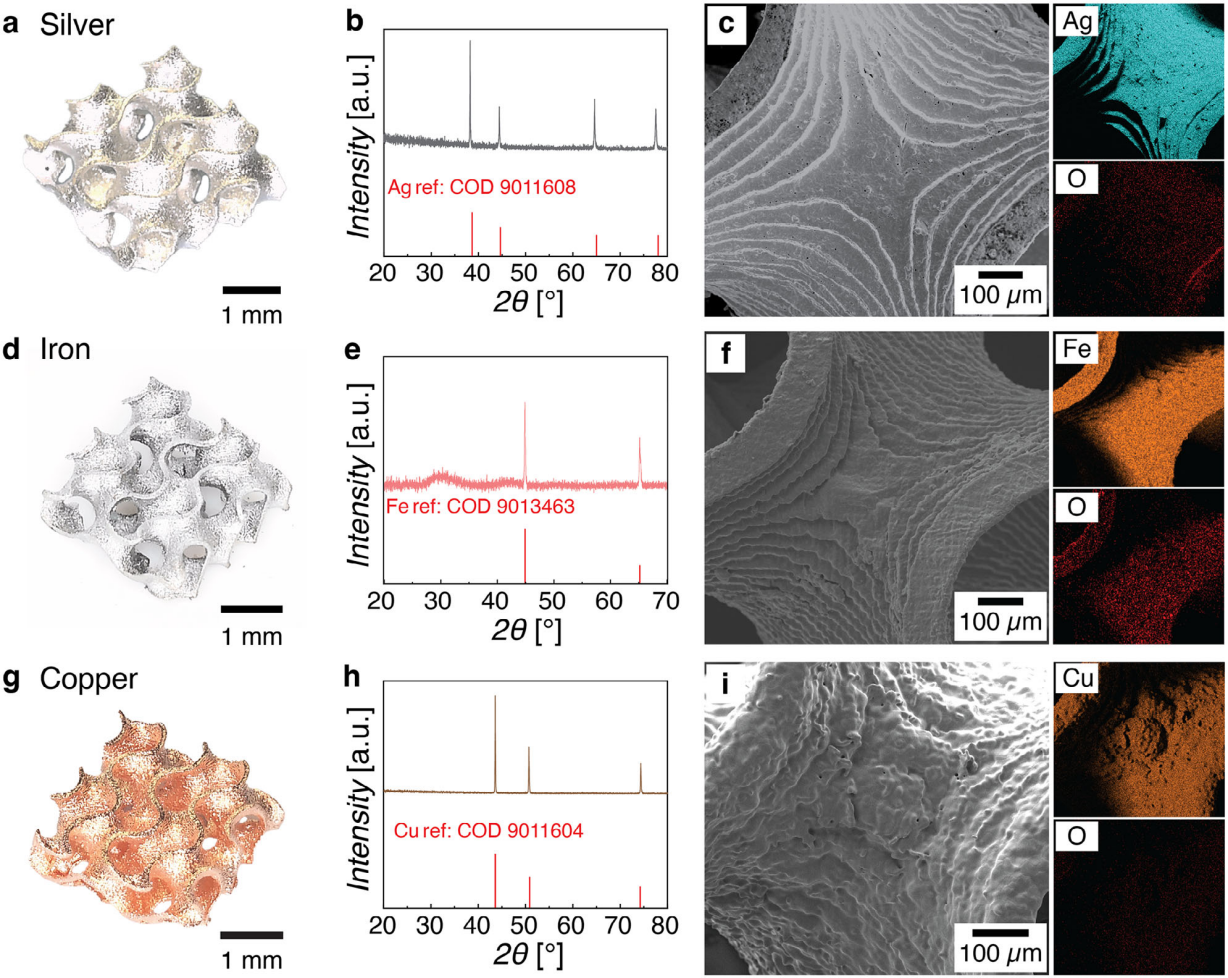
a,d,g) Optical images, b,e,h) XRD patterns, and c,f,i) EDS elemental maps of a–c) silver, d–f) iron, and g–i) copper gyroids.

We then utilized a two‐step thermal treatment process—i) debinding in nitrogen, ii) sintering in air—to remove the polymer template and sinter the precipitated particles together. The two‐step process was chosen as it was previously shown to be effective in mitigating crack formation during thermal treatment.^[^ ^68^, ^69^ ^]^ To optimize the debinding and sintering process, we conducted thermogravimetric analysis (TGA) measurements on the dried composites to identify the key reaction stages in both steps of the process (Figures S12–S14, Supporting Information). The debinding and sintering parameters for the composites were then set according to the TGA data (Figures S12–S14, Supporting Information). X‐ray diffraction (XRD) patterns obtained from the structures post‐calcination revealed that the iron oxide and copper composites were converted to Fe_2_O_3_ and CuO respectively (Figure S15, Supporting Information). The silver composites were directly converted to Ag since AgO / Ag_2_O thermally decomposes to Ag above 400 °C^[^ ^70^ ^]^ (**Figure** **3**a,b) Importantly, the total overall mass loss from the debinding and sintering of the iron oxide, copper, and silver composites were 34%, 15%, and 21% respectively (Figures S12–S14, Supporting Information), which is significantly lower than what can be typically achieved with state of the art metal‐salt solution approaches. For comparison, CuO and Ag structures fabricated by Ma et al.^[^ ^56^ ^]^ and Saccone et al.^[^ ^50^ ^]^ respectively experienced a mass loss of approximately 75% and 65% respectively. Fe_2_O_3_ structures prepared from direct calcination of iron‐ion infused hydrogels, i.e., using the conventional HIAM approach,^[^ ^50^ ^]^ underwent a mass loss of approximately 84% (Figures S16 and S17, Supporting Information). These improvements in retained mass led to significant reductions in part shrinkages—the Fe_2_O_3_, CuO, and Ag gyroid structures were approximately 20%, 31%, and 37%, respectively, smaller than the original as‐printed “blanks” (Figure S18, Supporting Information). Shrinkage was evaluated relative to the as‐printed, undeveloped structures since that best reflects the shrinkage of the process — from printed hydrogel to final material.

The Fe_2_O_3_ and CuO gyroid structures were then thermally treated in forming gas (95% N_2_, 5% H_2_,) to reduce them to Fe and Cu respectively (Figure 3e,h). The reduced metal structures (Fe, Cu, and Ag) all appeared shiny and maintained their overall gyroid architecture (Figure 3a,d,g). Taken together, the overall shrinkages from the as‐printed “blank” hydrogels to the Fe and Cu structures were approximately 38% and 46% respectively. To accurately evaluate the success of our shrinkage reduction strategy, it is necessary to take into consideration the density of our material as well, since a structure that undergoes minimal dimensional shrinkage but remains highly porous (e.g., a shell) will have little mechanical integrity. One approach to estimating density is to compare the experimentally observed linear shrinkage with the theoretical linear shrinkage that would occur based on the mass changes observed and assuming full conversion of the metal oxide to metal (Discussion S3, Supporting Information). From the TGA data in Figures S12–S14 (Supporting Information), the theoretical densities of the Fe, Cu, and Ag structures were determined to be 88%, 84%, and 84% respectively. To corroborate these density calculations, the microstructures of the Fe, Cu, and Ag structures were measured using X‐ray microcomputed tomography (µCT), and their reconstructed volumes used to experimentally determine their densities.^[^ ^71^ ^]^ The experimental densities of the Fe, Cu, and Ag structures were 91%, 88%, and 76% respectively, which were similar to that determined from the theoretical calculations. The comparatively lower experimental densities of the Ag structures were likely due to the strong X‐ray attenuation properties of Ag itself, which made µCT imaging inaccurate (see Discussion S4, Supporting Information). Consistent with the density calculations, the Fe microstructures did not contain any large pores or cracks (Figure S19, Supporting Information). Micropores could be observed in the µCT scans, but we were unable to accurately measure their sizes due to the insufficient resolution of our scans. However, some microporosity was expected given the theoretical density of 88.0%. The µCT scans of the Cu gyroids revealed a larger degree of porosity and cracking (Figure S20, Supporting Information), which is consistent with the lower calculated theoretical densities. The low shrinkages and comparatively high densities (Table S2 and Discussion S5, Supporting Information) of our metal structures is testimony to the effectiveness of our infusion‐precipitation strategy for the VP of metal.

The dimensional fidelities of the fabricated metal parts were evaluated by comparing their reconstructed µCT scans with scaled down versions of their CAD models (Figure S1, Supporting Information). Using CloudCompare (an open‐source software), the µCT reconstructions were aligned to the CAD models using six correlated points, and the difference between them determined. As shown in Figure S21 (Supporting Information), the mismatch distances for the iron, copper, and silver gyroids were 9 ± 42 µm, –3 ± 48 µm, and 18 ± 56 µm, respectively. To highlight the improved fidelity afforded by our infusion‐precipitation technology, we examined Fe gyroids fabricated using the HIAM method and found substantially larger mismatch distances of 43 ± 54 µm. These results confirm that the reduced shrinkage associated with our infusion‐precipitation process also reduced warpage. Further analysis of the mismatch distance heat maps revealed that the largest mismatch distances were mostly observed at the edges of the structures. To quantify this edge effect, we excluded the outer 12.5% of the structure (by linear length) from all directions and recalculated their mean mismatch distances. Without the edge contributions, the mismatch distances for the inner core regions of iron, copper, and silver gyroids were reduced to 2 ± 37, 1 ± 38, and 2 ± 38 µm, respectively. The mismatch distances for the HIAM Fe gyroids even without edge contributions were still higher at 33 ± 41 µm (Figure S21f, Supporting Information). Taken together, these findings demonstrate that our infusion‐precipitation process yields structures with improved dimensional fidelities across the bulk of the structure, with the largest mismatches confined to their periphery.

EDS elemental maps of the metal structures confirmed a uniform distribution of the main metal throughout the structure (Figure 3c,f,i and Figure S22, Supporting Information). A trace amount of oxygen was detected, likely due to oxidation of the surface to metal oxide. High levels of carbon (≈15–28 at% depending on the metal) were detected and were likely from combustion residue and/or contamination. These could potentially be reduced in future by pre‐treating the debinded structures in oxygen plasma prior to sintering and reduction.^[^ ^72^ ^]^ Nevertheless, the EDS and XRD data combined (Figure 3) suggested that our thermal treatments were successful in completely converting the composites to metals. SEM imaging of the metal gyroids revealed smooth surfaces with wall thicknesses of approximately 77–90 µm, depending on the composition. As compared to the wall thickness of the original “blank” hydrogel (150 µm), the wall thicknesses of the metal structures were consistent with the overall linear shrinkages of the structures. Additional SEM images and average grain sizes of the metals can be found in Figure S23 (Supporting Information). Some macroscopic cracks were observed on the surface of the structures—these cracks occurred either from the thermal conversion process and/or from handling of the materials. A challenge with any ceramic/metal VP process is that damage accumulates over the entire process, which then manifests itself as cracks or other macroscale structural damage in the final structure. As mentioned earlier, we observed that our composites were the most susceptible to damage. Sufficiently large scratches on the composite often led to the formation of cracks during the thermal conversion processes. However, with proper handling, we were able to achieve an average yield of ≈90% from printed hydrogels to final metal parts. The design of more mechanically robust and/or self‐healing hydrogels would help reduce handling sensitivity and improve the yield, but it is a subject of future work.

It is important to note that our infusion‐precipitation process allows us to achieve larger wall thicknesses in our metal structures while still maintaining high material densities. In most studies that utilize the metal‐salt approach for ceramic/metal fabrication, attention has always been drawn to their ability to achieve small feature sizes by exploiting shrinkage, i.e., shrinkage is portrayed as an advantage. However, shrinkage is a double‐edged sword in that it also imposes limitations on the maximum achievable feature sizes. This drawback is hardly discussed even though it fundamentally limits the types of parts that can be made and, thus, the practical applications of the technology. Although the wall thickness of the metal structures can be increased by starting with proportionally larger polymer templates to compensate for the anticipated shrinkage, this strategy has its limits. Beyond a certain critical polymer thickness, shrinkage cannot be controlled well as the gases evolved during the debinding process are unable to effectively escape from the structure due to the large diffusion distances. This results in the formation of highly porous, or even shell‐like, ceramics/metals with poor mechanical properties. Since shrinkage and density are often coupled, the reduced material density often manifests itself as a reduction in shrinkage, which can be misleading if shrinkage alone is used as a metric of success. Since the thermal conversion shrinkages are lower with our infusion‐precipitation‐based method, higher metal wall thicknesses can now be achieved for the same critical polymer thickness. We expect this ability to fabricate dense components with larger wall thicknesses will expand the range of practical parts that can be produced using metal‐salt VP technology.

### 2.4 Mechanically Robust Parts

**Figure 4:**
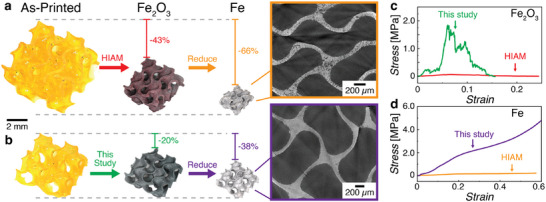
Comparison of density and shrinkage between Fe_2_O_3_ and Fe gyroids made using a) HIAM and b) our infusion‐precipitation approach. Representative engineering stress‐strain curves of the c) Fe_2_O_3_, and d) Fe gyroid lattices under compression.

To highlight the marked improvement of our infusion‐precipitation‐based process over the state of the art, we compared our Fe_2_O_3_ and Fe gyroids against their counterparts fabricated using the HIAM process.^[^ ^50^ ^]^ Given the large mass losses with the HIAM of Fe_2_O_3_ (Figure S16, Supporting Information), we found that we had to increase the size of the “blank” hydrogel structure by 20% to obtain Fe_2_O_3_ structures that were comparable in size to ones made with our infusion‐precipitation process (**Figure** **4**a,b). At these template sizes, the “blank”‐to‐ceramic linear shrinkage for the HIAM process was approximately 43% (Figure 4a). µCT scans and SEM images of the HIAM Fe_2_O_3_ structures revealed significant internal cracking and porosity (Figure S24, Supporting Information), which is consistent with the calculated theoretical density of 22% (Discussion S6, Supporting Information). The low densities likely arose due to the larger templates needed to achieve the same wall thicknesses as the structures made via the infusion‐precipitation method (vide supra).

The Fe_2_O_3_ structures were then compressed to determine their mechanical properties (Figure 4c and Figure S25, Supporting Information). As expected, the porous and heavily cracked HIAM‐made structures were only able to sustain a maximum engineering stress of ≈0.1 MPa. In contrast the denser structures fabricated with our infusion‐precipitation method could withstand significantly higher engineering stresses of approximately 2 MPa. Although the Fe_2_O_3_ structures were similar in size and shape, they were noticeably different after reduction in forming gas. The HIAM Fe_2_O_3_ structures shrank by 40% during the reduction process, for a total “blank”‐to‐metal linear shrinkage of 66% (Figure 4a). For comparison, the “blank”‐to‐metal linear shrinkage for the infusion‐precipitation process was 38%. The calculated theoretical and experimental density of the HIAM Fe structures were ≈49% and ≈50% respectively (Discussions S4 and S6, Supporting Information). µCT scans of Fe structures made via the infusion‐precipitation method confirmed that they were significantly denser than their HIAM counterparts (Figure 4a,b). As expected, the Fe gyroids fabricated using our approach exhibited markedly improved mechanical performance, with compressive strengths reaching ≈5 MPa compared to only ≈0.2 MPa for the HIAM‐derived structures, i.e., a ≈25‐fold increase (Figure 4d, Figure S25, Supporting Information). Nanoindentation experiments performed on the struts of the infusion‐precipitation Fe gyroid structures revealed the hardness and stiffness to be ≈178 Hv and 81 GPa respectively. The HIAM‐prepared Fe structures were too porous to be indented meaningfully (Figure S26, Supporting Information). This substantial enhancement in strength, clearly underscores the utility and advantage of our technology in enabling the fabrication of mechanically robust parts. Additional mechanical characterization and discussion of the metal structures fabricated using the infusion‐precipitation approach can be found in Discussion S7 (Supporting Information).

### 2.5 Demonstrating Scalability and Application Versatility

**Figure 5:**
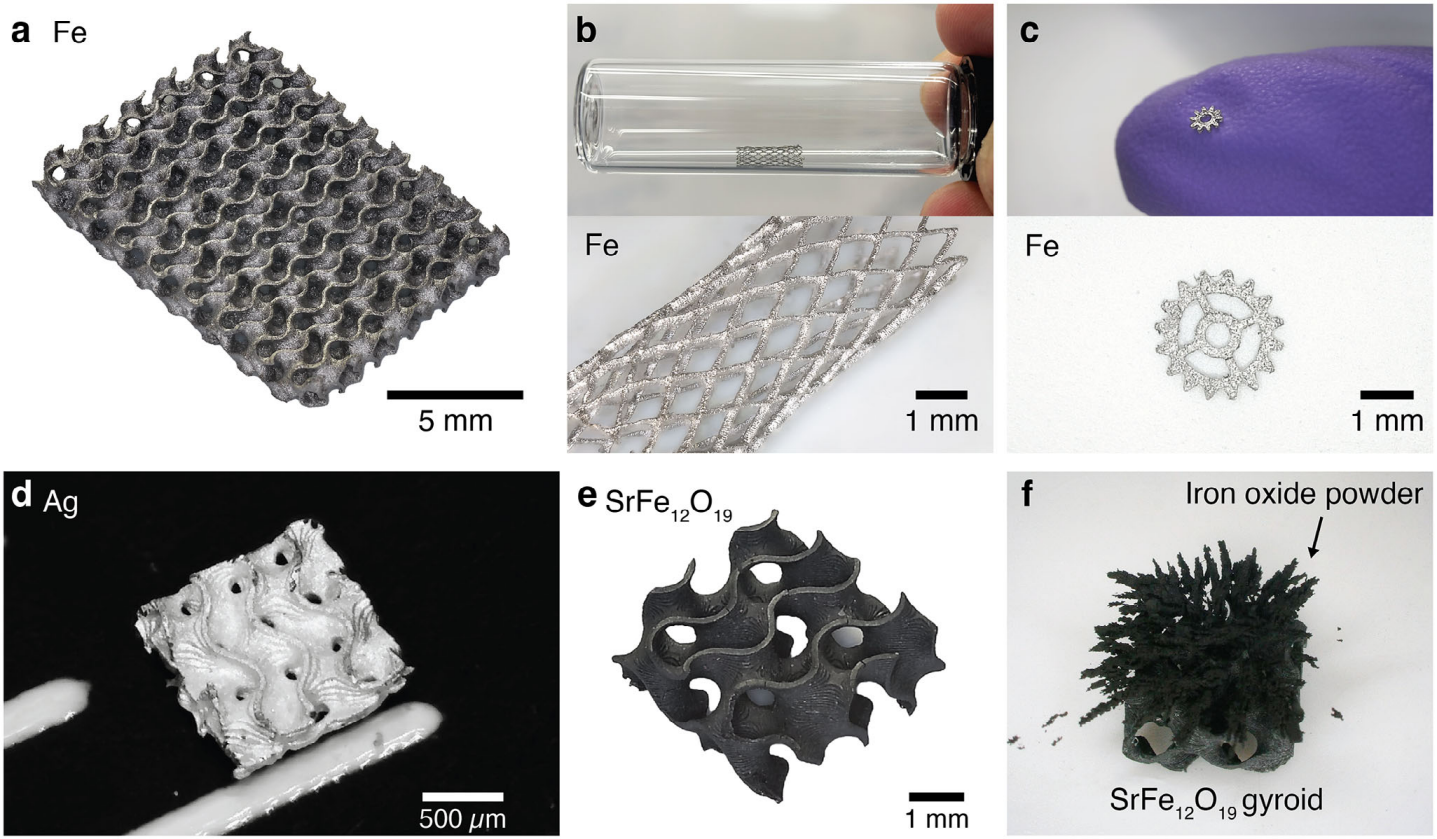
Optical images of centimeter‐scale Fe. a) gyroid lattice and b) stent, and c) millimeter‐scale Fe gears fabricated using our infusion‐precipitation approach. d) Optical image of millimeter‐scale Ag gyroid. e) Optical image of the SrFe_12_O_19_ gyroid structures. f) Iron oxide powder was used to visualize the magnetic field around the SrFe_12_O_19_ structure.

Most metal‐salt‐based metal printing approaches struggle with scalability since their large polymer‐to‐metal shrinkages requires the use of impractically large polymer templates. In addition, the significant shrinkages of these large templates are frequently accompanied by significant warping, which limit the utility of the final metal parts. To highlight the improved scalability of our infusion‐precipitation approach, we fabricated centimeter‐scale Fe gyroid lattices ≈ 1.3 × 1.0 cm in size, with beam sizes ≈ 90 µm (**Figure** **5**a). As seen from Figure S27 (Supporting Information), the size of the polymer template needed for our process is significantly reduced compared to the state of the art (≈60% shrinkage). Importantly, the lattices remained nearly flat after thermal conversion, demonstrating the ability of our method to produce large metal architectures with excellent part fidelities.

The reduced warping achieved with our process broadens the range of parts that can be fabricated. While warping can sometimes be tolerated in lattice structures, it becomes highly detrimental in planar or tubular structures. To illustrate this, Fe gears (planar) and stents (tubular) fabricated with our infusion‐precipitation process retained their intended geometries (Figure 5b,c), whereas those produced with the original HIAM method exhibited severe deformation (Figure S28, Supporting Information). These stents and gears are representative of critical components used in biomedical and mechanical devices, underscoring the immediate potential of our technology for manufacturing non‐lattice‐based industrial‐relevant parts.

It is important to emphasize that the infusion‐precipitation process developed in this study is complementary to established metal fabrication methods such as selective laser sintering (SLS). SLS can easily achieve centimeter‐to‐meter scale metal parts with >1 mm wall thicknesses, which our technology is unable to achieve at the moment. However, achieving metal structures with wall thicknesses below 300 µm is generally difficult to achieve with SLS, with only a few advanced systems reaching ≈100 µm.^[^ ^73^ ^]^ This challenge is further amplified for highly reflective metals such as copper^[^ ^74^ ^]^ and silver,^[^ ^75^ ^]^ which absorb laser energy less efficiently; an issue that our fabrication process avoids entirely. Although the silver and copper structures in Figure 3 already exhibited <100 µm wall thicknesses, we fabricated additional silver gyroids with ≈30 µm wall thicknesses (Figure 5d)—which is beyond the practical limits of SLS today—to further highlight the capabilities of our technology. The mean mismatch distance of this smaller silver gyroid structure from the scaled CAD file was 33 ± 37 µm. The differences in mismatch distance between the silver gyroids in Figures 5d and 3a were likely due to the sizes of the polymer template used (see Discussion S8, Supporting Information). Finally, SLS technology is currently compatible with a broader range of metals compared to metal‐salt processes, as it is not limited to metals that can be readily reduced by forming gas. However, this versatility comes at a significantly higher cost due to the requirement for expensive lasers, atmosphere control, and powder handling systems. In contrast, our infusion–precipitation approach enables the fabrication of metals using only standard VP systems, metal salts, and a tube furnace, offering a more cost‐effective and accessible alternative to metal fabrication.

Beyond structural metal parts, our technology can also be applied to fabricate functional ceramics. To demonstrate this, we fabricated 3D gyroid structures out of hard‐magnetic strontium hexaferrite (Figure 5e; Figures S29 and S30, Supporting Information). This was achieved by simply infusing the iron oxide composite structure with a solution of strontium salt prior to thermal treatment, enabling the formation of SrFe_12_O_19_ through an in situ reaction and conversion. This highlights the modularity of our process, where different functional compositions can be accessed by tuning only the final infusion step. Vibrating sample magnetometry (VSM) showed that the SrFe_12_O_19_ structures exhibited hard magnetic behavior and a magnetization of ≈60 emu/g at 15 kOe (Figure S30, Supporting Information), which is consistent with the magnetic properties of bulk SrFe_12_O_19_. A simple iron oxide fillings test was used to visually demonstrate the hard magnetic behavior of our 3D structure (Figure 5f). Considering the critical role of hard magnets in multiple areas of science and engineering, and the growing interest in non‐rare‐earth sustainable magnets, our ability to fabricate architected hard‐magnetic ceramics via our infusion‐precipitation route opens new opportunities for integrating magnetic functionality into complex 3D architectures.

**Figure 6:**
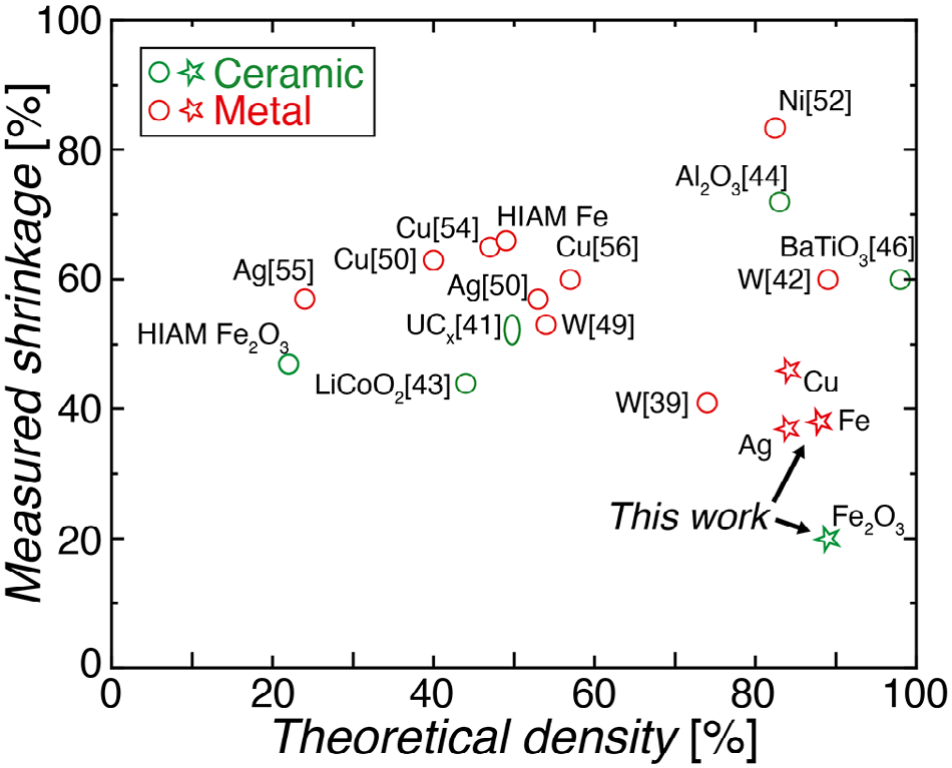
Comparison of linear shrinkages and theoretical densities for this work and other relevant metal‐salt solution VP works. Our infusion‐precipitation‐based strategy enables the fabrication of ceramics and metals with higher densities and lower shrinkages than the state of the art. See Table S2 and Discussion S5 (Supporting Information) for the calculation of the theoretical shrinkages.

Finally, to benchmark our technology against the state of the art, we compared our reported shrinkage and theoretical density values against other relevant works in this area (**Figure** **6** and Table S2 and Discussion S5, Supporting Information). The selected works cover a variety of methods that utilize metal‐salts as a source of metal precursors, including infusion‐based processes,^[^ ^50^, ^51^, ^52^, ^54^, ^55^, ^56^ ^]^ general metal‐salt‐containing resins,^[^ ^39^, ^42^, ^43^, ^44^, ^47^, ^48^, ^57^ ^]^ and sol‐gel processes.^[^ ^41^, ^46^ ^]^ The data in Figure 6 demonstrates that the process developed in this work is capable of producing architected ceramic and metal structures that have shrinkages and densities that significantly surpass the state of the art.

## 3 Conclusion

In summary, we developed a facile and versatile method for the VP of high‐density ceramics and metals with low conversion shrinkages. Central to our approach is the post‐fabrication chemical conversion of our “blank” hydrogels into hydrogel composites with high metal loadings. We achieve this via a repeated infusion‐precipitation process that progressively synthesizes metal‐containing particles in situ within the hydrogels. The hydrogel composites are then thermally converted into ceramics or metals. With our infusion‐precipitation method, we retain all the advantages of the metal salt solution method while decoupling hydrogel metal loading from its ion equilibrium adsorption capacity. We fabricated hydrogel templates with equivalent metal‐ion loadings > 50 wt% and used them to make Fe, Cu, and Ag structures with linear shrinkages of 38%, 46%, and 37% respectively, and theoretical densities of 88%, 84%, and 84% respectively. Importantly, parts made using our technology are significantly more robust and scalable than the state of the art, which broadens the range of potential real‐world applications. Although this study only demonstrated the fabrication of Fe, Cu, Ag, Fe_2_O_3_, and SrFe_12_O_19_, we expect the technology to be amendable to a wider variety of ceramics and metals since we can readily leverage the extensive library of nanoparticle synthesis methods developed by the inorganic chemistry community. We expect our technology to be compatible with other AM technologies that utilizes gels, and will be particularly beneficial for technologies that cannot tolerate particle‐induced light‐scattering, such as with volumetric additive manufacturing^[^ ^76^ ^]^ and two‐photon lithography (TPL). For example, in TPL, the requirement for optically transparent resins significantly limits the incorporation of metal particles, which in turn restricts metal loading and leads to substantial shrinkage during thermal conversion. Our infusion–precipitation strategy overcomes this limitation, potentially enabling the low shrinkage TPL of dense metal structures with sub‐micron features, which is highly challenging to achieve today. While not the focus of this study, our infusion‐precipitation approach could also unlock the TPL of composites with high filler weight fractions, which is impossible to achieve today. Moving forward, we expect that automation of the repetitive infusion and precipitation process will reduce the labor associated with the current manual process and also mitigate some of the challenges with hydrogel handling. The improved part robustness, scalability, and feature sizes possible with our technology will likely open up new avenues for scientific exploration that were previously impossible or highly challenging. For example, the ability to fabricate larger, high‐fidelity architected structures is essential for studying fluid flow and mass transport in porous media, investigating mechanical behavior in composites with architected fillers, validating scaling laws in mechanical metamaterials, and developing functional devices such as architected carbon capture or catalytic systems. More importantly, this ability to fabricate high‐quality functional and structural architected materials could enable future studies into architecture‐property relationships and the development of novel 3D devices, i.e., this work lays the foundation for a fabrication platform that can be translated to applications well beyond the scope of this study.

## 4 Experimental Section

### Materials

Tartrazine (≥85%, Sigma‐Aldrich), iron (III) chloride hexahydrate (FeCl_3_ • 6H_2_O, 97%, Sigma‐Aldrich), iron (II) chloride tetrahydrate (FeCl_2_ • 4H_2_O, 98%, Sigma‐Aldrich), copper (II) nitrate trihydrate (Cu(NO_3_)_2_ • 3 H_2_O, 98.0–103% (RT), Sigma‐Aldrich), silver nitrate (AgNO_3_, cryst. extra pure, Sigma‐Aldrich), sodium borohydride (NaBH_4_, ≥ 98%, Sigma‐Aldrich), strontium chloride hexahydrate (SrCl_2_ • 6H_2_O, 99%, Sigma‐Aldrich), ammonium hydroxide solution (28.0–30.0% for analysis EMSURE ACS, Reag. Ph Eur, Sigma‐Aldrich), poly(ethylene glycol) diacrylate *M*_n_ = 700 g mol^−1^ (PEGda 700, Sigma‐Aldrich), lithium phenyl‐2,4,6‐trimethylbenzoylphosphinate (LAP) (≥95%, Sigma‐Aldrich), and iron (II, III) oxide powder (Fe_3_O_4_, powder, < 5 µm, 98%, Sigma‐Aldrich) were used as received without further purification. The iron (II, III) oxide powder was used for the SrFe_12_O_19_ demonstration in Figure 5f.

### Methods: “Blank” Hydrogel Resin Formulation

To prepare the “blank” hydrogel photoresin, 20 mg of LAP and 15 mg of tartrazine were first dissolved in deionized water to give a solution of volume 5 mL. 5 mL of PEGda 700 was then added and mixed until a homogenous transparent solution was formed.

### Additive Manufacturing via DLP Printing

Additive manufacturing was performed using a commercial multi‐wavelength DLP printer (MONO3‐MZ4, MonoPrinter). The design of the gyroid lattice structures printed in this study is shown in Figure S1 (Supporting Information). The exposure time was set at 30 s for the base layers and 16 s for the rest.

### Development of Printed Structures

After fabrication, the hydrogel lattices were yellow in color due to the presence of tartrazine. The as‐printed structures were soaked in deionized water for 15 mins at room temperature to remove any unreacted photoresin components in and around the structure. The deionized water was then decanted, and the hydrogels soaked again in fresh deionized water for 1 h at room temperature. At this point, most of the tartrazine in the hydrogels were removed, and the structures appeared colorless. The hydrogels were then soaked again in deionized water overnight, before being removed and stored in fresh deionized water at room temperature.

### Preparation of Metal Salt Solution

The iron salt infusion solutions were prepared by dissolving FeCl_3_ · 6H_2_O and FeCl_2_ · 4H_2_O in a 1.8:1 mol ratio in deionized water. To prepare the 1.5 m Fe^2+^ and 2.7 m Fe^3+^ infusion solution used for Fe_2_O_3_ and Fe structure fabrication, 7.298 g of FeCl_3_ · 6H_2_O and 2.982 g of FeCl_2_ · 4H_2_O were dissolved in deionized water and the solution made to 10 mL. The copper salt infusion solutions were prepared by dissolving Cu(NO_3_)_2_ · 3H_2_O in deionized water. To prepare the 5 m copper infusion solution used for CuO and Cu fabrication, 12.08 g of Cu(NO_3_)_2_ · 3H_2_O was dissolved in deionized water and the solution was made to 10 mL. The silver salt infusion solutions were prepared by dissolving AgNO_3_ in deionized water. To prepare the 10 m solution used for Ag fabrication, 8.5 g of AgNO_3_ was dissolved in deionized water and the solution was made to 5 mL.

### Sample Handling

Hydrogel structures were placed in a cage, and the cage moved between the infusion and precipitation solutions. This minimized direct handling of the hydrogels. The hydrogel composites were only removed from the cage prior to the drying process. A plastic spatula was carefully used to avoid applying excessive pressure onto the composites.

### Metal‐Ion Infusion

The hydrogel or hydrogel composite structures were placed in metal salt infusion solutions with the desired composition and concentration of metal salts. The infusion solutions were heated to the desired temperatures using a hot plate. The structures were left to soak in the solution without any stirring. After infusing for the desired time, the cages that contain the metal ion‐infused hydrogels were removed from the solution and dabbed dry with a paper towel to absorb any salt solution trapped in the structures and on their surface. The infusion conditions for all metal salt solutions were 65 °C for 60 min.

### In Situ Coprecipitation of Iron Oxide

The ammonium hydroxide solution was first poured into a glass vial at room temperature. The iron‐infused hydrogels were then placed into the ammonium hydroxide solution for some time. The orange‐brown iron‐infused hydrogels turned black in a few seconds on contact with the ammonium hydroxide solution. After reacting for the desired amount of time, the iron oxide composites were removed from the ammonium hydroxide solution and washed 3 times with deionized water. The structures were then ultrasonicated in water for 30 s to remove any excess surface‐bound iron oxide. Following that, the structures were soaked in fresh deionized water for 1 hour before further processing. Throughout the entire process, the hydrogels and hydrogel composites were kept hydrated to minimize dimensional changes. For the fabrication of iron oxide composites, the in situ coprecipitation conditions were 5 min in the ammonia solution at room temperature.

### In Situ Reduction of Ag

A 1 m sodium borohydride solution was first prepared by dissolving the appropriate amount of sodium borohydride in ice‐cold deionized water. The sodium borohydride solutions were always prepared fresh and used immediately. The silver‐infused hydrogels were then placed into 10 mL of the ice‐cold 1 m sodium borohydride solution for the desired amount of time. The yellow silver‐infused hydrogels turned silver within a few seconds of contact with the sodium borohydride solution. After reacting for the desired amount of time, the Ag composites were removed from the sodium borohydride solution and washed 3 times with deionized water. The structures were then ultrasonicated in water for 30 s to remove any excess surface‐bound Ag particles. Following that, the structures were stored in fresh deionized water before further processing. Throughout the entire process, the hydrogels or hydrogel composites were kept hydrated to minimize dimensional changes. For the fabrication of Ag composites, the in situ reduction time used was 5 min.

### In Situ Reduction of Cu

The protocol for the in situ reduction of Cu is identical to that of the Ag except that the concentration of sodium borohydride solution used was 0.25 m.

### Strontium Ferrite Preparation

The strontium‐ion solution was prepared by dissolving 6 g of strontium chloride hexahydrate in deionized water and the solution made to 10 mL solution. The 10‐cycle iron oxide composites were then immersed in the solution at 65 °C overnight. The drying and thermal treatments of strontium‐infused 10‐cycle iron oxide composites were the same as that of the iron‐infused iron oxide composites.

### Mass Measurements

Samples were first dried at room temperature overnight and then further dried on a hot plate at 65 °C for 1 h. The dried samples were then placed in a weighing boat and weighed using an analytical balance. The mass of at least 3 samples were measured for each processing condition tested.

### Drying Prior to Thermal Processing

Samples were dried at room temperature overnight in the presence of silica gel.

### Tracking of Particle Growth within 3D Printed Pillar

Pillars 350 µm in diameter and 3 mm tall were printed between two 0.2 mm thick base plates. The pillars were then immersed in the appropriate metal salt solution at 65 °C for 1 h. The ion‐infused pillars were then immersed in a glass vial containing the precipitating solution for a period of time. Post‐precipitation, the pillars were removed from the glass vials and dabbed dry with a paper towel. The pillars were laid flat on the paper towel and sliced using a blade. The sliced pillars were then immediately stored in a glass vial filled with deionized water to prevent dehydration. The pillars were then imaged with an optical microscope (Keyence VHX 6000) to determine the extent of precipitation within the hydrogel.

### Cross‐Section Imaging of 3D Gyroid Composites

The gyroid composites were first dried at room temperature with silica‐gel and then embedded in PDMS overnight. The PDMS‐embedded samples were then bisected and then coated with gold prior to SEM imaging.

### Material Characterization

The samples were imaged with optical microscopy (MRCL 700, Microqubic AG and Keyence VHX 6000) and scanning electron microscopy (SEM) (GEMINI 2, Zeiss AG, 5 kV). The particle size distribution was calculated by using ImageJ StarDist on the SEM images. Energy‐dispersive X‐ray spectroscopy (EDS) maps and line‐scans were obtained with a Zeiss Crossbeam 540 equipped with an Oxford Inst. Ultim Max EDX detector. The applied voltage used during EDS was 15 kV. Powder X‐ray diffraction (XRD, D6 PHASER, Bruker) patterns were collected at 40 kV and 15 mA using a Cu source with a LYNXEYE XE‐T detector. The ceramic powders were obtained by pulverizing the respective composites using a mortar and pestle. The metal films were obtained by manual flattening using wooden boards. The microstructures of the printed structures except for silver were characterized by X‐ray computed tomography (EasyTom L, RX Solutions) at 80 kV and 80 µA. The µCT scans of the silver structures were obtained at 100 kV and 70 µA. A copper filter was placed in front of the X‐ray tube to absorb low energy X‐ray. The sliced µCT scan images and reconstructed 3D clouds were exported from xact64 (software, RX Solutions). Fidelity data (mismatch distance) was calculated by using “Cloud to map” function in CloudCompare (open‐source software, Ver. 2.13.2). For these calculations, the reconstructed CT scan models were manually aligned to their scaled down CAD file via 6 sets of correlated points. Compression tests of the ceramics and metals were conducted on a Zwick Z020 with a 1 kN load cell. The compression strain rate was 0.1 min^−1^ for metals and 0.05 min^−1^ for ceramics. Nanoindentation was performed on polished samples using an Anton Paar NHT2 nanoindenter with diamond Berkovich tip (B‐U 09). The parameters of all indentation tests were set as: 10 Hz acquisition rate, linear loading, 50 mN max load, 50 mN min^−1^ loading rate, 50 mN min^−1^ unloading rate, and 30 s pause. The polishing procedure used was as such: the metal gyroids were first embedded in epoxy and then sequentially polished in a polisher using 6, 3, and 1 µm diamond polishing liquids to obtain smooth metal cross‐sections. Vibrating sample magnetometer (VSM, Quantum Design PPMS, DynaCool) was employed to investigate the magnetic properties. The VSM measurements were conducted at 300 K and at a pressure of 26 Torr. Thermal treatments were conducted in a tube furnace (MTI, OTF‐1200X‐S) with a gas flow controller (EF‐FLOW, Bronkhorst). The heating programs are listed in Figures S12–S14 (Supporting Information). The average grain sizes of the metals were determined from the SEM images using ImageJ and the linear intercept method.

## Conflict of Interest

The authors declare no conflict of interest.

## Supporting information



Supporting Information

## Data Availability

The data that support the findings of this study are available from the corresponding author upon reasonable request.
