# Supplementary material for: Hydrogel‐Based Vat Photopolymerization of Ceramics and Metals with Low Shrinkages via Repeated Infusion Precipitation
Source: Adv Mater. 2025 Sep 24;38(8):e04951. doi: 10.1002/adma.202504951 (PMC12878802; doi:10.1002/adma.202504951)
Supplement: Supplementary file 1 — Supporting Information [file ADMA-38-e04951-s001.pdf]

ADVANCED MATERIALS

Supporting Information

for *Adv. Mater.*, DOI 10.1002/adma.202504951

Hydrogel-Based Vat Photopolymerization of Ceramics and Metals with Low Shrinkages via Repeated Infusion Precipitation

*Yiming Ji, Ying Hong, Dhruv R. Bhandari and Daryl W. Yee**

Supporting Information

Hydrogel-Based Vat Photopolymerization of Ceramics and Metals with Low Shrinkages via Repeated Infusion-Precipitation

*Yiming Ji, Ying Hong, Dhruv R. Bhandari, and Daryl W. Yee**

Y. Ji, Y. Hong, D. R. Bhandari, D. W. Yee
Institute of Electrical and Micro Engineering
École Polytechnique Fédérale de Lausanne (EPFL)
Rue de la Maladière 71b, Neuchâtel 2000, Switzerland.

D. R. Bhandari
Department of Metallurgical Engineering & Materials Science
Indian Institute of Technology Bombay
Main Gate Rd, IIT Area, Powai, Mumbai, Maharashtra 400076, India.

E-mail: daryl.yee@epfl.ch

Supplementary Figures

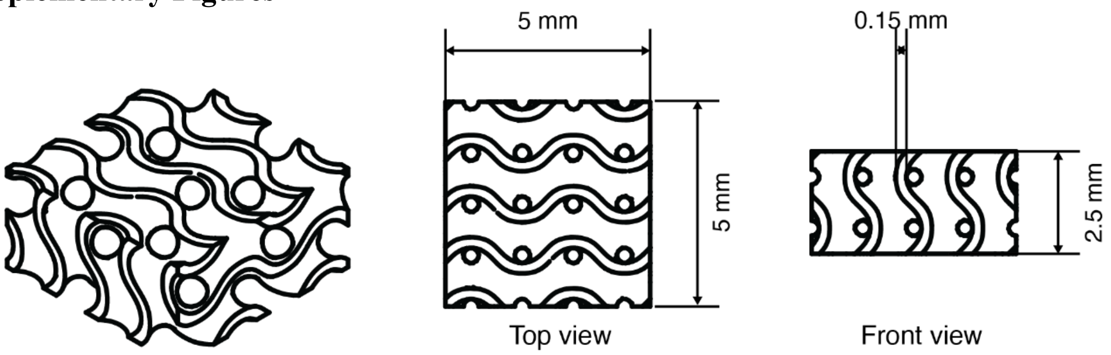

Figure S1. Design of gyroid structure. The gyroid structures have beam thicknesses of 150 μm, width = length = 5 mm, and height = 2.5 mm.

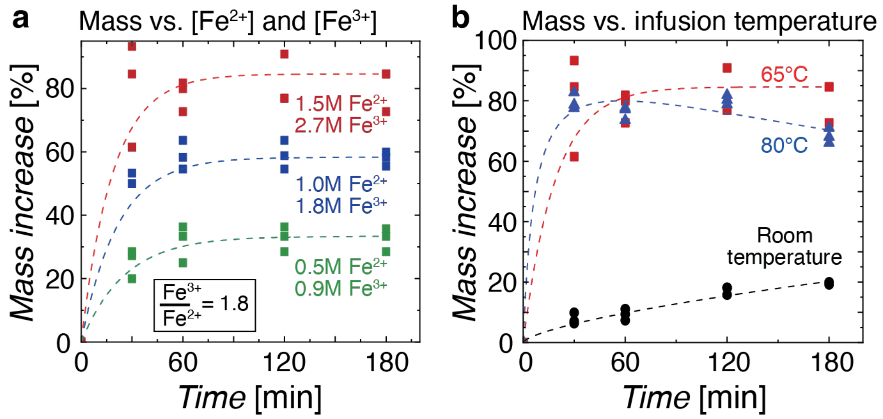

Figure S2. a) Mass increase of the hydrogel after infusion as a function of time and iron salt concentration. The infusion temperature was kept constant at 65˚C. b) Mass increase of the hydrogel after infusion as a function of time and temperature. The concentration of salts used were 1.5M Fe^{2+} and 2.7M Fe^{3+}.

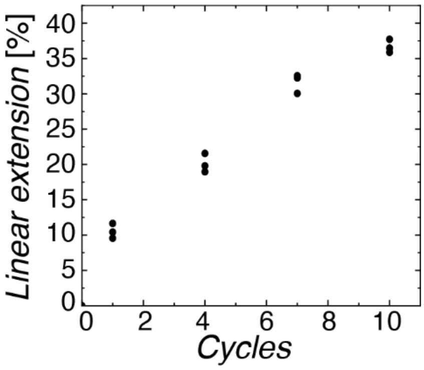

Figure S3. Linear expansion of iron oxide composite as a function of infusion-coprecipitation cycles.

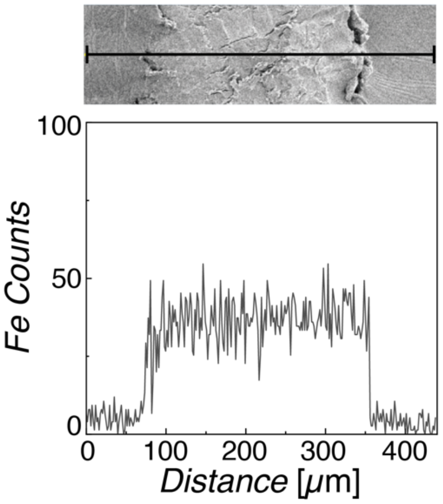

Figure S4. EDS Fe line scans of cross-sections of 10-cycle iron oxide composites. Fe was detected throughout the cross-section of the structure. The composites were embedded in PDMS prior to cross-sectioning. The regions without Fe are PDMS.

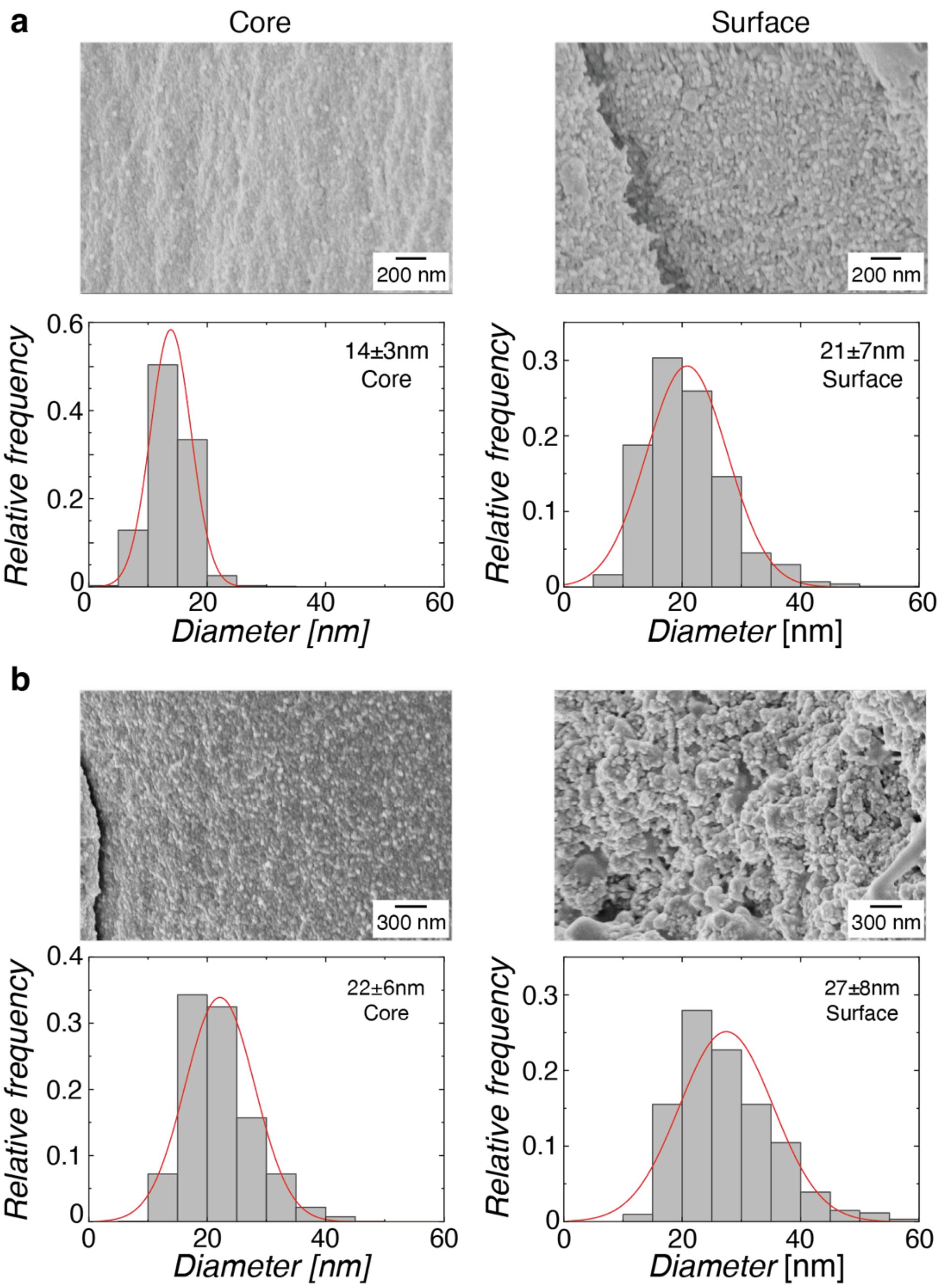

Figure S5. Histogram of iron oxide particle sizes measured from SEM images taken from either the core or surface of cross-sections of a) 7-cycle and b) 10-cycle composites. Left column: core; Right column: surface.

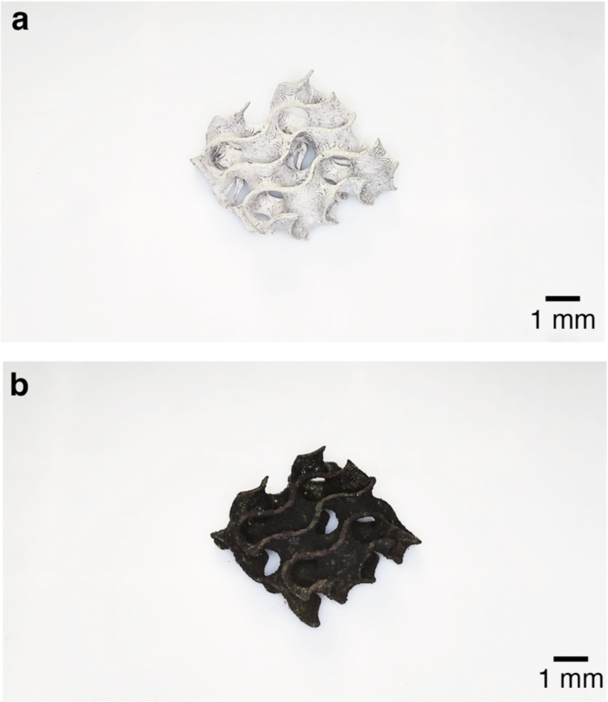

Figure S6. Optical image of the dried a) 5-cycle Ag composite and b) 7-cycle Cu composite. The black color of the Cu composite is likely due to oxidation.

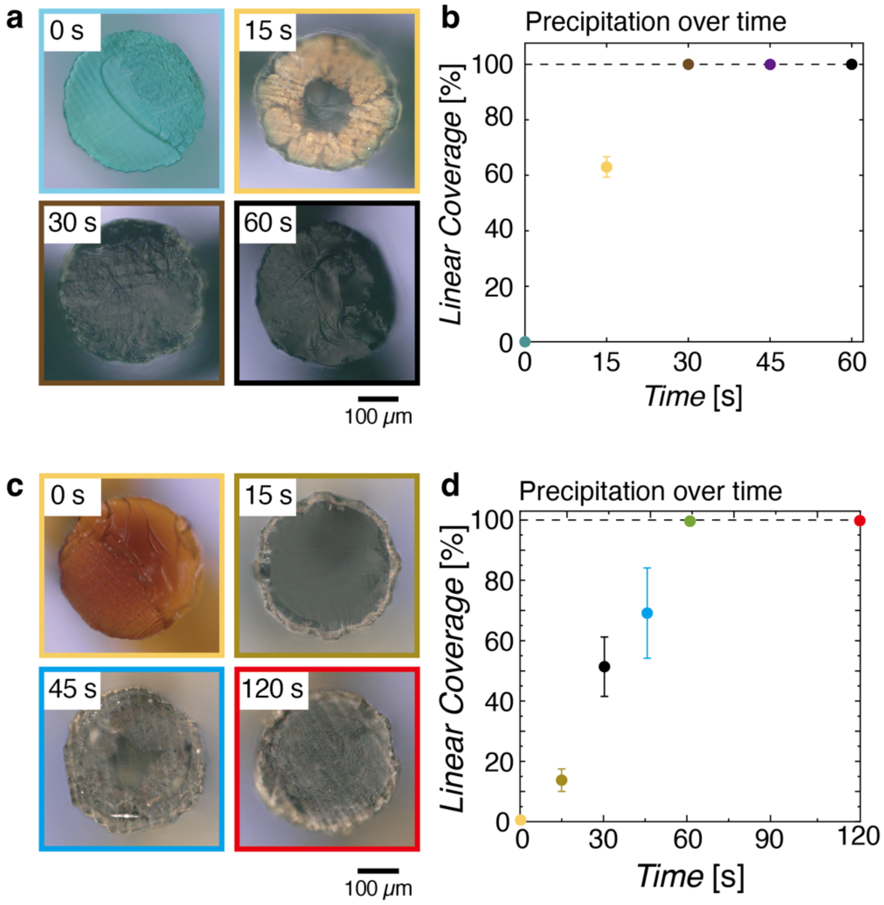

Figure S7. Cross-sections of a) Cu-infused and c) Ag-infused hydrogel pillars after different exposure times to sodium borohydride solution. b) Extent of *in-situ* reduction as a function of sodium borohydride exposure time of b) Cu-infused and d) Ag-infused hydrogel pillars. Linear coverage is measured by the length of the growth front as compared to the radius of the pillar.

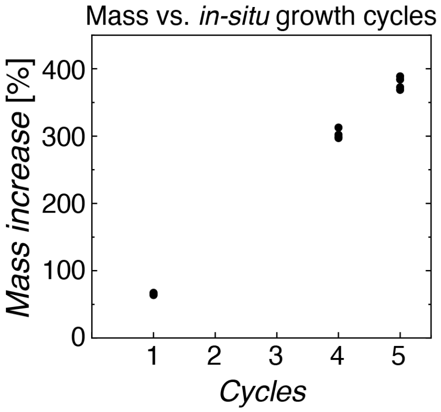

Figure S8. Mass increase of the hydrogel as a function of the number of Ag infusion-precipitation cycles.

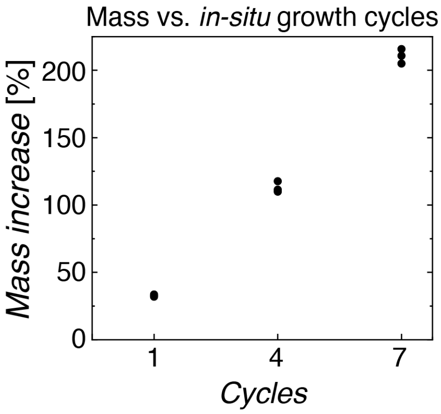

Figure S9. Mass increase of the hydrogel as a function of the number of Cu infusion-precipitation cycles.

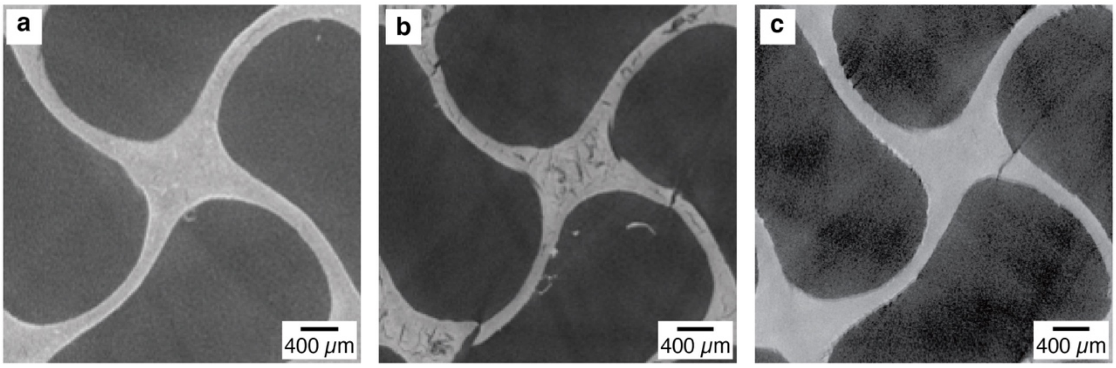

Figure S10. μCT scan of a) hydrated, b) rapidly dried, and c) RT-dried with silica gel 10-cycle infused iron oxide composite.

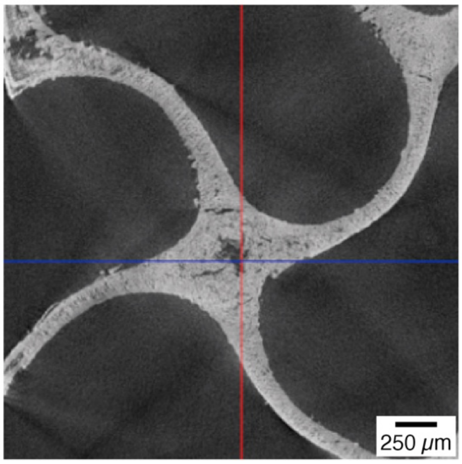

Figure S11. μCT scan of a Fe_2O_3 structure made from the thermal treatment of a rapidly dried 10-cycle iron oxide composite. The internal cracks formed in the composite after rapid drying were carried over to the Fe_2O_3 structure. Slow drying of the composite is thus necessary to prevent the formation of internal cracks.

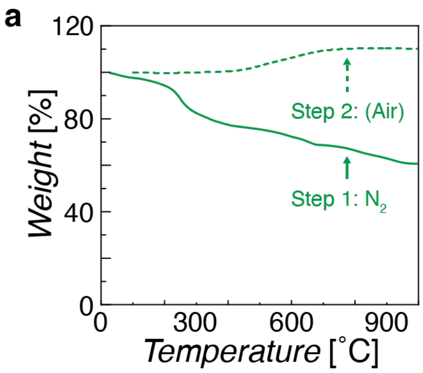

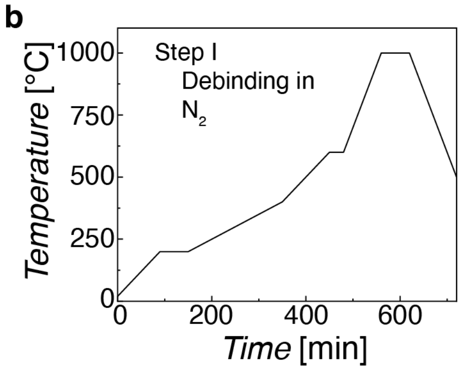

Step I		
Temperature (°C)	Duration (min)	Heating rate (°C/min)
20-200	90	2
200	30	-
200-350	600	0.25
350-1000	325	2
1000	30	-

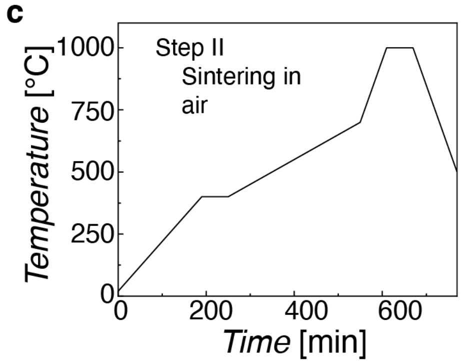

Step II		
Temperature (°C)	Duration (min)	Heating rate (°C/min)
20-400	190	2
400-700	300	1
700-1000	150	2
1000	30	-

Figure S12. a) TGA profiles of the infused 10-cycle iron oxide composites in nitrogen (solid line) and then in air (dashed line). Heating profiles used for b) debinding (N_2) and c) sintering (air) of the infused 10-cycle iron oxide composites.

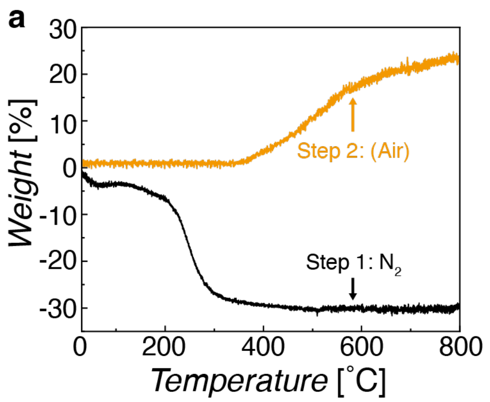

b

Step I		
Temperature (°C)	Duration (min)	Heating rate (°C/min)
20-200	90	2
200	30	-
200-350	600	0.25
350-600	125	2
600	30	-

c

Step II		
Temperature (°C)	Duration (min)	Heating rate (°C/min)
20-300	140	2
300-700	400	1
700-800	50	2
800	30	-

Figure S13. a) TGA profiles of the 7-cycle Cu composites in nitrogen (black line) and then in air (orange line). Heating profiles used for b) debinding (N_2) and c) sintering (air) of the 7-cycle Cu composites.

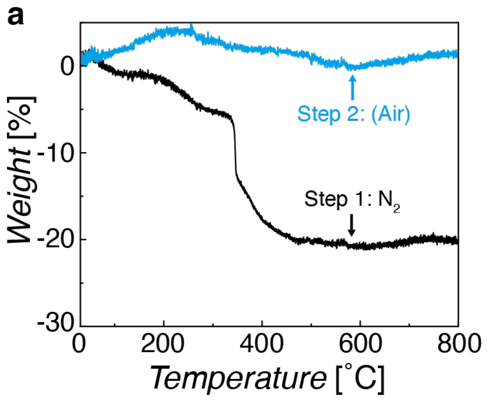

b

Step I		
Temperature (°C)	Duration (min)	Heating rate (°C/min)
20-200	90	2
200	30	-
200-550	1400	0.25
550-700	75	2
700	30	-

c

Step II		
Temperature (°C)	Duration (min)	Heating rate (°C/min)
20-700	340	2
700	30	-

Figure S14. a) TGA profiles of the 5-cycle Ag composites in nitrogen (black line) and then in air (blue line). Heating profiles used for b) debinding (N_2) and c) sintering (air) of the 5-cycle Ag composites.

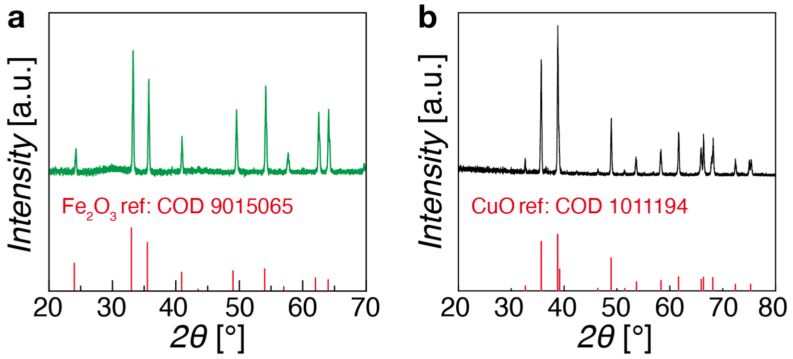

Figure S15. XRD patterns of the a) infused 10-cycle iron oxide composite and b) 7-cycle copper composite after debinding and sintering.

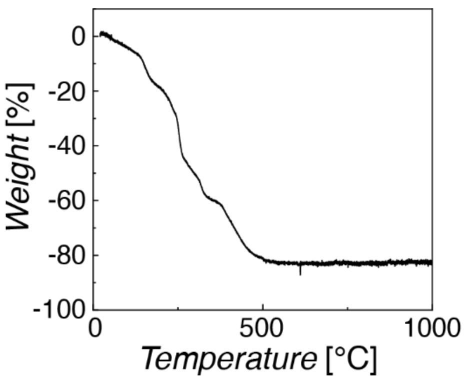

Figure S16. TGA data obtained from direct calcination of an Fe-ion infused hydrogel (HIAM process). A mass loss of over 80% is observed.

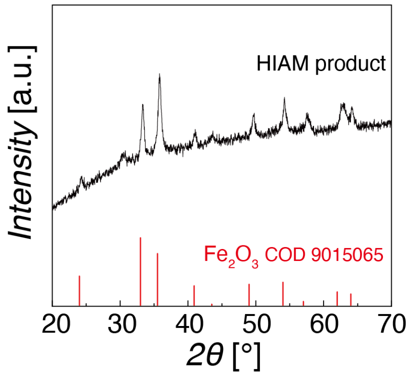

Figure S17. XRD pattern of an Fe-ion infused hydrogel (HIAM process) after calcination in air at 1000˚C.

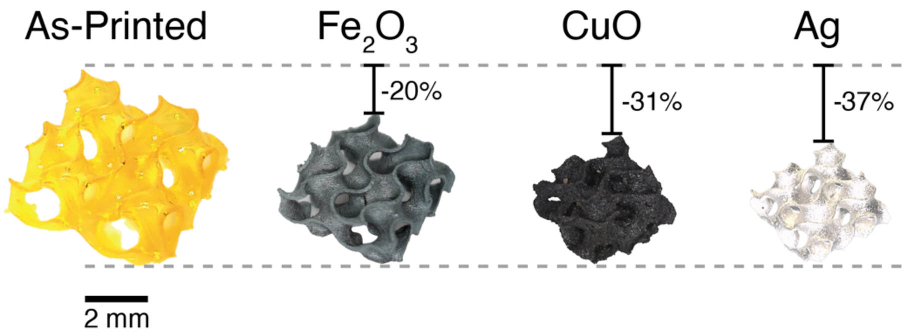

Figure S18. Optical images of "Blank" as-printed hydrogel vs. the fabricated Fe_2O_3, CuO, and Ag structures. The Fe_2O_3, CuO, and Ag structures were obtained after debinding and sintering of the iron oxide, copper, and silver composites respectively.

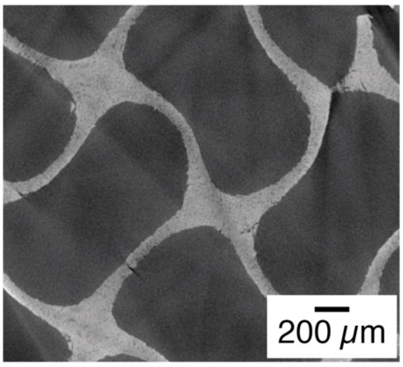

Figure S19. μCT scan of a Fe structure made from an infused 10-cycle iron oxide composite.

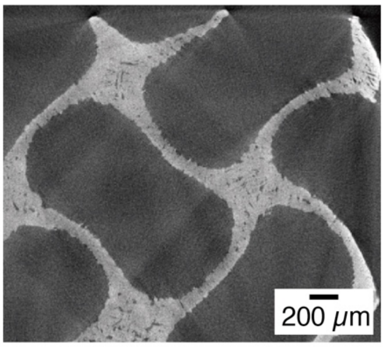

Figure S20. μCT scan of a Cu structure made from a 7-cycle Cu composite.

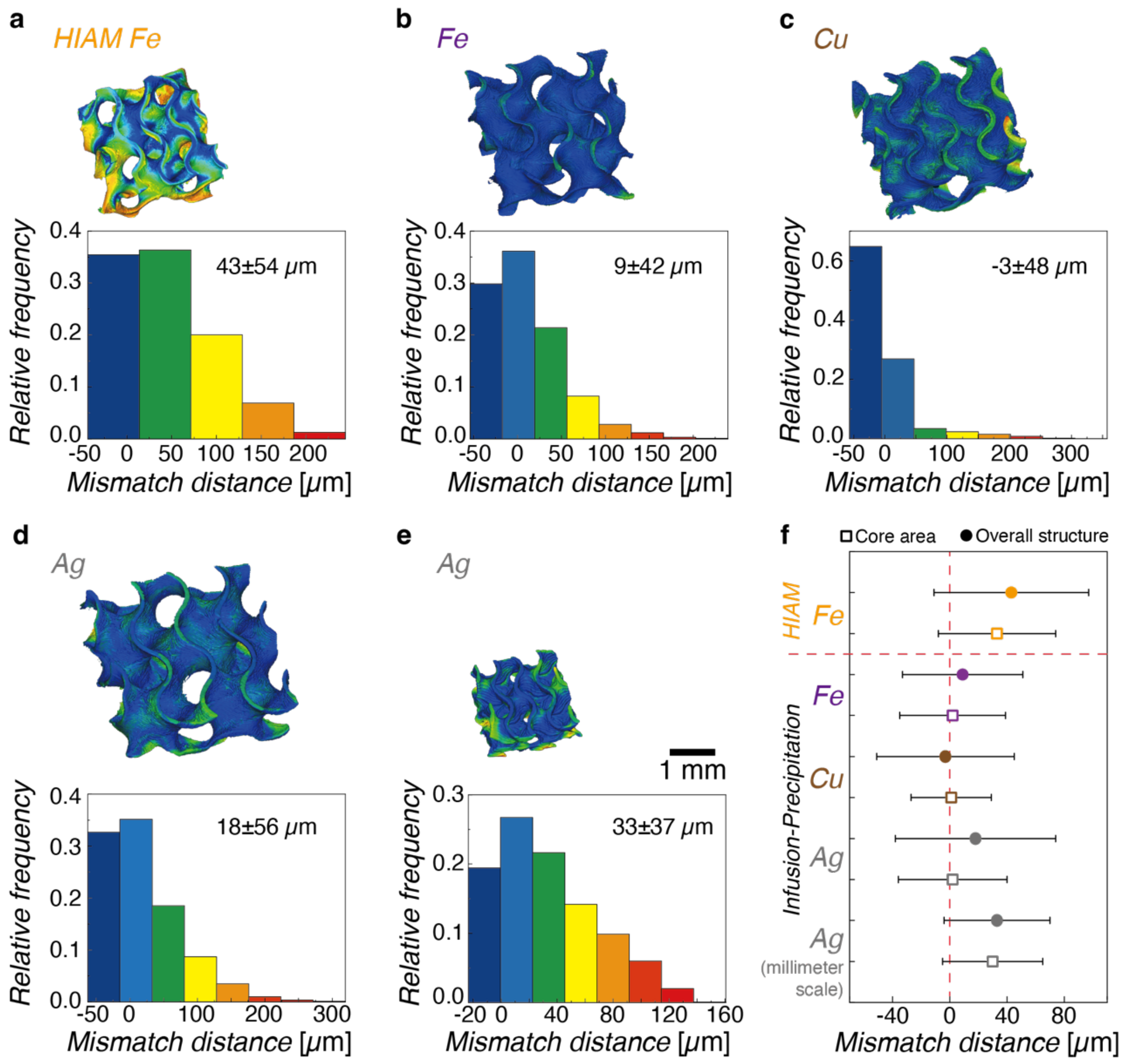

Figure S21. Mismatch heat map and histogram of gyroid lattices. a) Iron prepared with HIAM method. b) Iron, c) copper, and d,e) silver prepared with the infusion-precipitation method. The scale bar of 1mm is relevant for all panels. f) Comparative analysis of mismatch distances between the overall structure and their core regions for the metals examined.

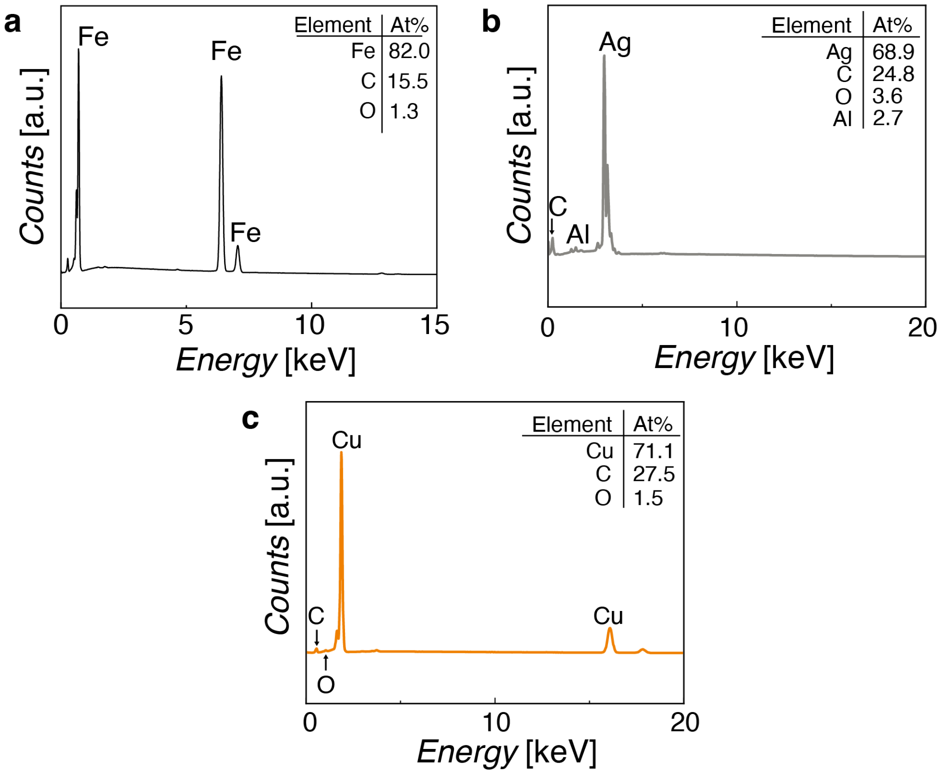

Figure S22. EDS spectrum of a) Fe, b) Ag, and c) Cu structures fabricated using our infusion-precipitation approach. A trace amount of oxygen was detected, likely due to oxidation of the surface to metal oxide. High levels of carbon (~ 15 - 28 at% depending on the metal) were detected and were likely from combustion residue and/or contamination.

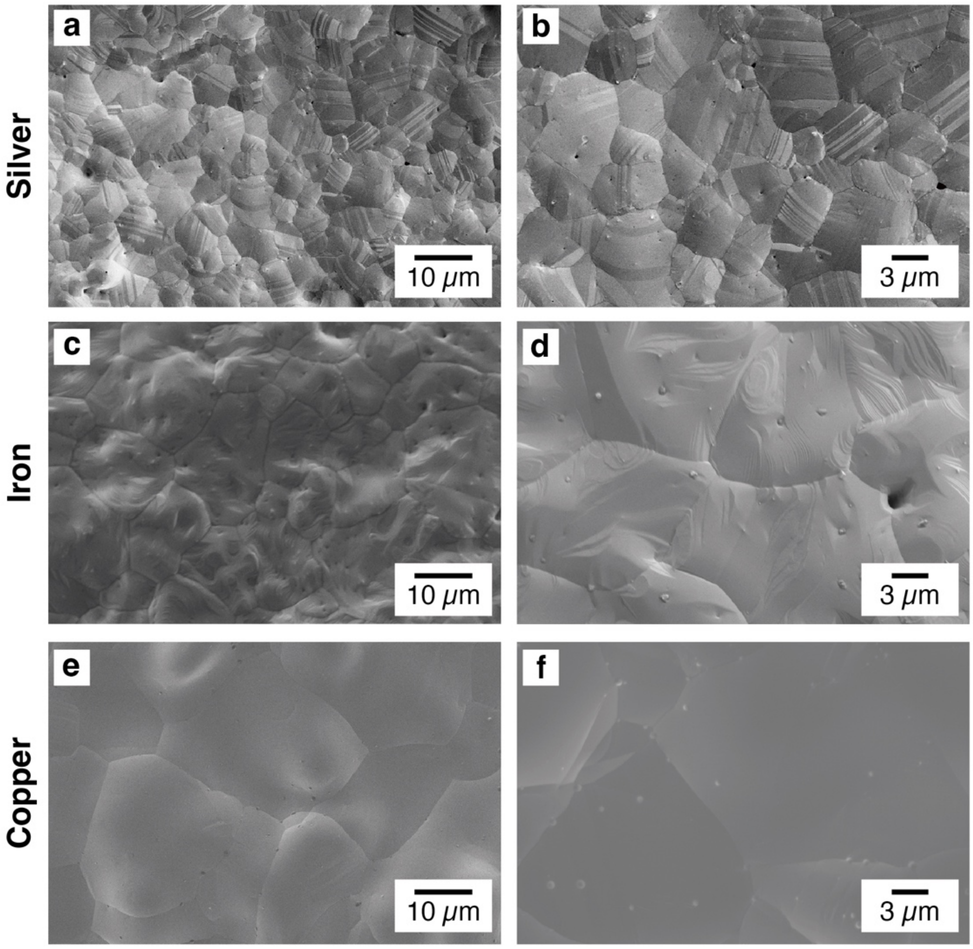

Figure S23. SEM images showing the microstructures of the a-b) Ag, c-d) Fe, and e-f) Cu gyroid structures. The average grain sizes of the Ag, Fe, and Cu structures are 4.1 ± 0.6 µm, 11.5 ± 2.0 µm, and 13.8 ± 3.5 µm respectively. The grain sizes were measured using the linear intercept method.

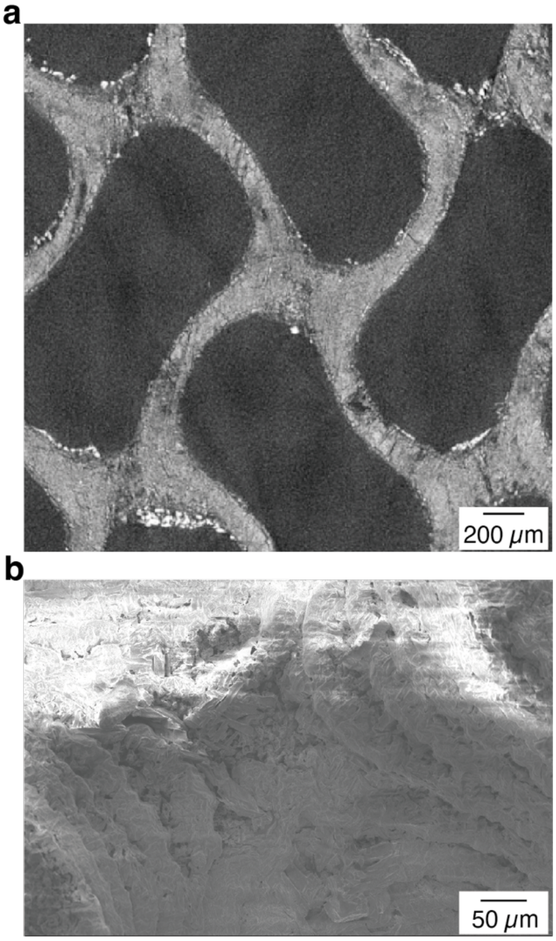

Figure S24. a) μCT scan and b) SEM image of Fe_2O_3 structures prepared using the HIAM process. The presence of large pores and cracks can be observed in both images.

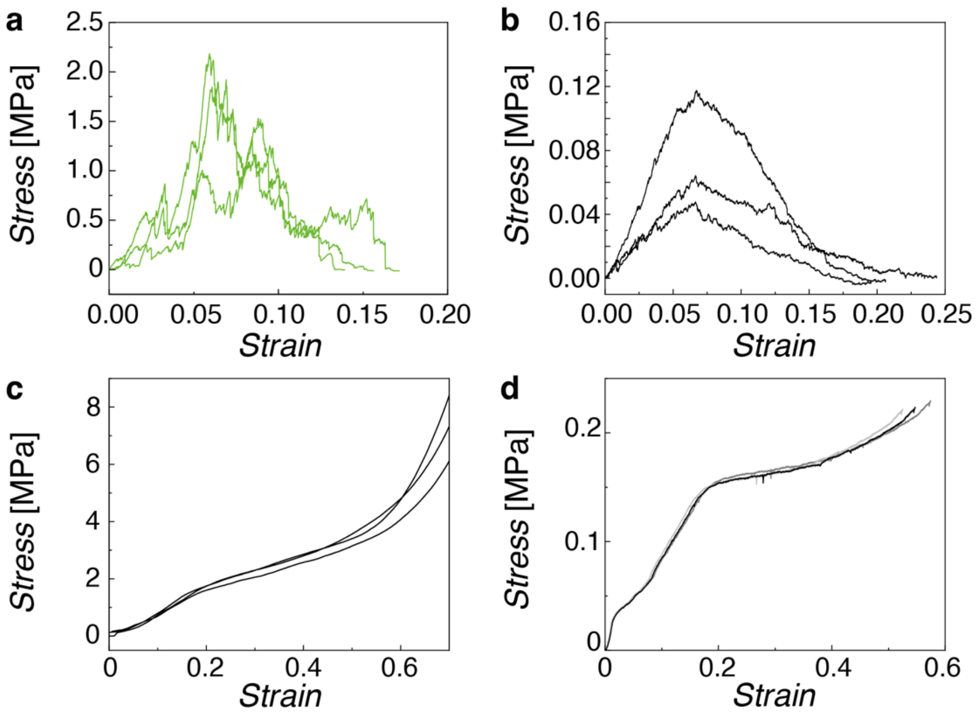

Figure S25. Engineering stress-strain plots of Fe_2O_3 structures fabricated via a) the infusion-precipitation method and b) the HIAM process. Engineering stress-strain plots of Fe structures fabricated via c) the infusion-precipitation method and d) the HIAM process. All structures were tested under compression. 3 structures of each were tested.

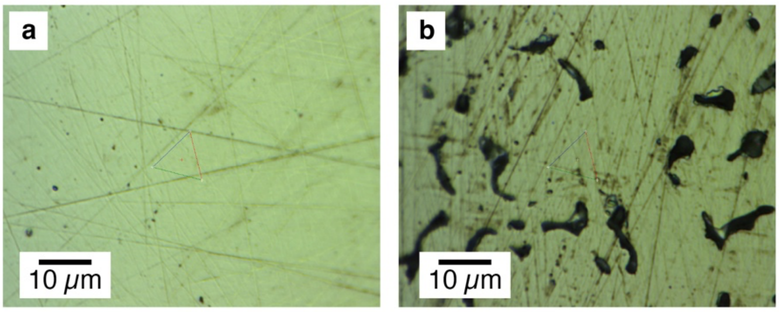

Figure S26. Optical images of polished surfaces of Fe gyroids prepared via a) infusion-precipitation and b) the HIAM process. The structures prepared via HIAM were too porous to be indented meaningfully.

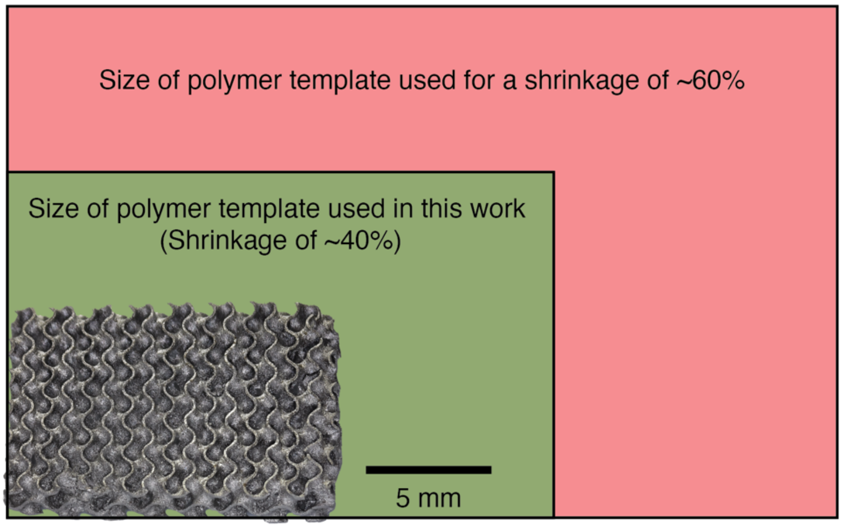

Figure S27. Size of polymer templates needed to fabricate the centimeter-scale Fe gyroid lattice shown in Figure 5a using our infusion-precipitation approach (40% shrinkage; green) and with other higher shrinkage approaches (60% shrinkage, red).

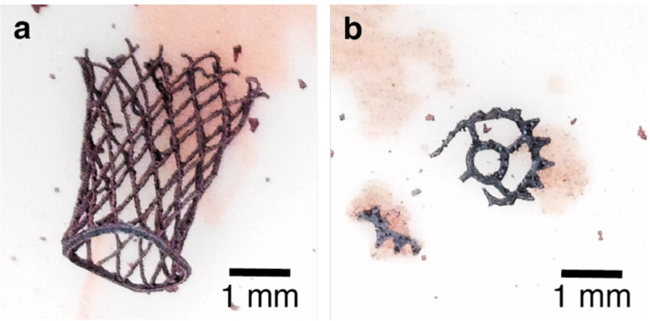

Figure S28. Fe_2O_3 a) stent and b) gear made via the HIAM method. The samples were not reduced to Fe since they were destroyed due to significant warping.

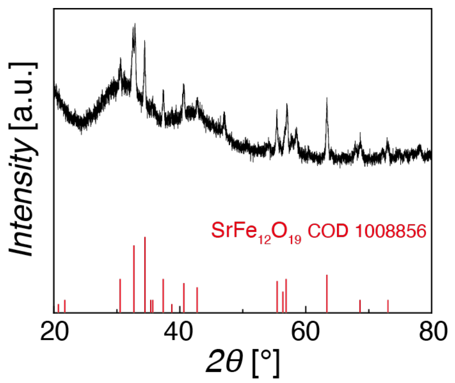

Figure S29. XRD pattern of the $SrFe_{12}O_{19}$ structures fabricated via the infusion-precipitation method. The amorphous hump around 30° is from the sample holder.

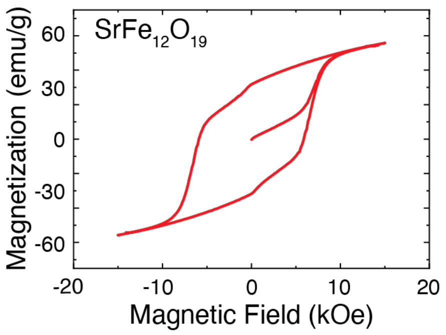

Figure S30. Hysteresis loop (measured by VSM, 300K) of the $SrFe_{12}O_{19}$ gyroid structures.

Supplementary Tables

Table S1. Hydrogel resin composition

Reagent	Purpose	Amount
Deionized water	Solvent	5 mL
Poly(ethylene glycol) diacrylate M_n = 700 g/mol (PEGda 700)	Binder	5 mL
Lithium phenyl-2,4,6-trimethylbenzoylphosphinate	Photoinitiator	20 mg
Tartrazine	UV blocker	15 mg

Table S2. Experimental shrinkage and theoretical density of materials reported in other relevant works

Study	Ref. in Main Text	Material	Experimental Shrinkage (%)	Theoretical density (%)
This work (Infusion-precipitation)	NA	Fe_2O_3	20	89
This work (Infusion-precipitation)	NA	Fe	38	88
This work (Infusion-precipitation)	NA	Ag	37	84
This work (Infusion-precipitation)	NA	Cu	46	84
This work (HIAM)	NA	Fe_2O_3	47	22
This work (HIAM)	NA	Fe	66	49
Saccone et al. [1]	50	Cu*	63	40
Saccone et al. [1]	50	Ag*	57	53
Martinez et al. [2]	54	Cu	65	47
Saigal et al. [3]	55	Ag	57	24
Ma et. al. [4]	56	Cu	60	57
Sun et. al. [5]	51	Fe**	55	NA
Zhang et. al. [6]	52	Ni	73 – 94	75 – 90
Yee et al. [7]	47	ZnO	70 – 90	NA
Yee et al. [8]	43	$LiCoO_2$	44	44
Cai et al. [9]	42	W	60	89
Hu et al. [10]	57	Variety	30 – 55	NA
Rosental et al. [11]	46	$BaTiO_3$	60	98
Dovdevany et al. [12]	44	Cr-doped Al_2O_3	72	83
Zanini et al. [13]	41	UC_x	50 – 55	50
Luitz et al. [14]	49	W	53	54
Zan et al. [15]	39	W	41	74

*Cu and Ag were used as a representative material for Saccone et al.
**Sun et al. sought to fabricate iron sponges and employed pore forming techniques.

[1] Saccone et al., *Nature* **2022**, *612*, 685.
[2] Martinez et al., *Adv. Manuf.* **2024**, DOI 10.1007/s40436-024-00514-z.
[3] Saigal et al., in *ASME 2023 Aerospace Structures, Structural Dynamics, and Materials Conference*, American Society Of Mechanical Engineers, San Diego, California, USA, **2023**, p. V001T03A007.
[4] Ma et al., *Angew Chem Int Ed* **2024**, *63*, e202405135.
[5] Sun et al., *Adv Funct Materials* **2024**, 2418035.
[6] Zhang et al., *Nano Lett.* **2023**, acs.nanolett.3c02309.
[7] Yee et al., *Adv. Mater.* **2019**, *31*, 1901345.
[8] Yee et al., *Adv Materials Technologies* **2021**, *6*, 2000791.
[9] Cai et al., *Advanced Science* **2024**, 2405487.
[10] Hu et al., *Advanced Materials* **2024**, *36*, 2405053.
[11] Rosental et al., *Chemical Engineering Journal* **2024**, *499*, 156189.
[12] Moshkovitz Douvdevany et al., *Journal of the European Ceramic Society* **2024**, *44*, 116773.
[13] Zanini et al., *Adv Funct Materials* **2024**, *34*, 2406916.
[14] Luitz et al., *Adv Eng Mater* **2023**, *25*, 2201927.
[15] Zan et al., *J. Phys. D: Appl. Phys.* **2022**, *55*, 444004.

Supplementary Discussion

Supplementary Discussion 1. Calculation of equivalent Fe loading in the 10-cycle iron oxide composites

From the experimental data shown in Figure 2c, the mass increase of the composite after 10 cycles of the infusion-coprecipitation process, as compared to the dried "blank" polymer, was 400%. The mass increase was calculated according to the formula shown below:

$$\boldsymbol{mass\ increase} = \frac{\boldsymbol{m_{10c} - m_{dbh}}}{\boldsymbol{m_{dbh}}} \times \mathbf{100}\ \%$$

Where m_{10c} is the mass of the dried composite and m_{dbh} is the mass of the dried "blank" hydrogel. For ease of calculation, let us assume that m_{10c} is 100 g and that the mass increase was entirely from the *in-situ* growth of Fe_3O_4. Accordingly, the mass of polymer and Fe_3O_4 in the composite is thus 20 g and 80 g respectively. Since the molar mass of Fe and Fe_3O_4 is 55.85 g/mol and 231.53 g/mol, respectively, the weight percent of Fe in Fe_3O_4 is thus:

$$\boldsymbol{wt\%_{Fe-in-Fe_3O_4}} = \frac{\mathbf{3 \times 55.85}\ \boldsymbol{g/mol}}{\mathbf{231.53}\ \boldsymbol{g/mol}} \times \mathbf{100}\ \boldsymbol{wt\%} = \mathbf{72.37}\ \boldsymbol{wt\%}$$

Thus, the equivalent mass of Fe in the dried 10-cycle composite is:

$$\boldsymbol{m_{Fe-ions-in-composite}} = \boldsymbol{wt\%_{Fe-in-Fe_3O_4}} \times \mathbf{80}\ \boldsymbol{g} = \mathbf{57.89}\boldsymbol{g}$$

The weight percent of Fe in the composite can thus be determined by:

$$\boldsymbol{wt\%_{Fe-ions-in-composite}} = \frac{\boldsymbol{m_{Fe-ions-in-composite}}}{\mathbf{100}\ \boldsymbol{g}} \times \mathbf{100}\ \boldsymbol{wt\%} = \mathbf{57.89}\ \boldsymbol{wt\%} \approx \mathbf{58\%}$$

The dried 10-cycle composites thus have an Fe loading of 58 wt%.

Supplementary Discussion 2: Increasing metal loading via an additional infusion step prior to thermal treatment.

To further increase metal loading, an additional infusion step prior to thermal treatment can be conducted. This was conducted for the iron system. We detail the calculations for the amount of iron in the infused 10-cycle iron oxide composites below. For simplicity, we assume that the particles grown were Fe_3O_4.

Calculation of equivalent Fe-ion loading in the infused 10-cycle IONP composites

To calculate the equivalent Fe loading in the Fe-ion infused 10-cycle IONP composites, we first need to determine the wt% of Fe in the infused Fe salts. The molar masses of $FeCl_2 \cdot 4H_2O$ and $FeCl_3 \cdot 6H_2O$ are 198.81 g/mol and 270.29 g/mol respectively. The ratio of $FeCl_2 \cdot 4H_2O$ to $FeCl_3 \cdot 6H_2O$ in our infusion solution was 1:1.8. For ease of calculation, the total mass of 1 mol of $FeCl_2 \cdot 4H_2O$ and 1.8 mol of $FeCl_3 \cdot 6H_2O$ is thus 685.33 g. The mass of Fe in 1 mol of $FeCl_2 \cdot 4H_2O$ and 1.8 mol of $FeCl_3 \cdot 6H_2O$ is thus (1 mol + 1.8 mol) x 55.85 g/mol = 156.38 g. The wt% of Fe in the amount of salts used is thus 156.38 g / 685.33 g x 100% = 22.82%.

We observed that the dried Fe-ion infused 10-cycle IONP composites were 27.14% heavier than the 10-cycle IONP composites. For ease of calculation, if we assume that the initial 10-cycle Fe_3O_4 composite was 100 g (similar to the example in Supplementary Discussion 1), the mass of the Fe-ion infused 10-cycle IONP composite is thus 127.14 g, i.e. the mass of infused salts is 27.14 g. We assume that the water of hydration is tightly bound to the metal ion and is not removed during drying. Of this 27.14 g of infused salts, the mass of Fe in them is 0.2282 x 27.14 g = 6.19 g. From Discussion 1, the mass of Fe in a 100 g dried 10-cycle Fe_3O_4 composite is 57.89 g. Thus, the total mass of Fe in the dried infused 10-cycle Fe_3O_4 composite is 57.89 g + 6.19 g = 64.08 g. The weight percent of Fe in the material is thus 64.08 g / 127.14 g x 100% = 50.4 %.

The dried Fe-ion infused 10-cycle IONP composites thus have an Fe loading of 50.4 wt%. Although the iron content appeared to decrease from 58 wt% to 50 wt% after the final infusion step, this was due to the additional mass contributed by the chloride counterions and water of hydration in the $FeCl_2 \cdot 4H_2O$ and $FeCl_3 \cdot 6H_2O$ salts. These species increased the overall mass of the composite, thereby diluting the measured wt% of iron, even though the absolute amount of iron in the structure actually increased.

To demonstrate the benefit of the infusion step, we compared the polymer-to-ceramic shrinkage of 10-cycle iron oxide composites with and without the final ion-infusion step (Figure S31). As can be seen from Figure S31, the samples without the final ion-infusion step shrank more than those that included it. Specifically, shrinkages of 20% and 35% were observed for the composites with and without the final infusion step respectively. The theoretical density of the structures without the final infusion was slightly higher at ~90% (compared to 88% for the ones with the final infusion).

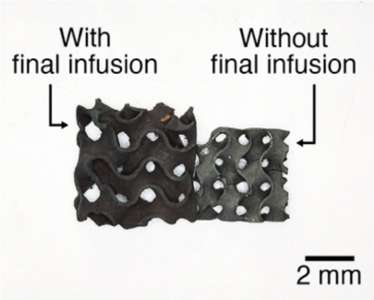

Figure S31. Optical images of Fe_2O_3 structures fabricated from 10-cycle iron-oxide composites with (left) and without (right) the final infusion step.

While the final infusion step can be used to increase the absolute mass of metal in the composite and reduce shrinkage, we also observed that it can negatively impact the density of the final ceramic or metal structure, depending on the type of metal salt used. In this study, we performed the final infusion using the same metal salt as in the preceding infusion-precipitation cycles to maintain compositional consistency and for operational simplicity. For the iron system, we used iron chlorides. However, for copper and silver, we utilized their respective metal nitrates.

We observed that, for both copper and silver, the final infusion with their metal nitrate solutions led to cracking during thermal treatment (Figure S32). We hypothesize that this is due to the strong oxidizing nature of nitrates, which can facilitate rapid gas evolution. The rapid gas evolution can lead to pressure buildup within the composite, which if not released adequately, can lead to cracking and/or porosity. As a result, we omitted the final infusion step for the copper and silver systems to preserve structural integrity.

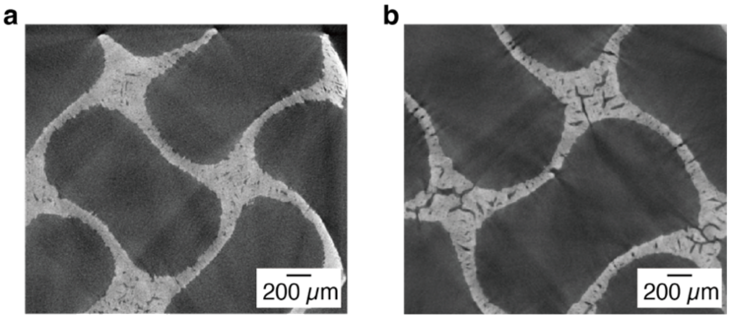

Figure S32. μCT scan of a Cu structure made from a 7-cycle Cu composite made a) without and b) with the final infusion with copper nitrate.

We posit that the use of less oxidizing metal salts could potentially allow for a final infusion in these cases; however, we anticipate that challenges in solubility could arise. For example, in the case of silver, silver salts with less oxidizing counter-ions, such as silver acetate, have significantly lower solubilities in water than their nitrate counterparts (almost 100x lower), which limits their utility as infusion solutions. Moving forward, optimizing the parameters of this final infusion step, including the composition and concentration of the salt solution used, will likely be necessary to utilize it effectively. However, this optimization lies beyond the scope of the current study. The key focus of this work is the use of repeated infusion–precipitation cycles to drastically increase the metal mass loading within the hydrogel scaffold. While the additional infusion step can further reduce shrinkage, the repeated infusion–precipitation strategy is still the main contributor to the improved densities and low shrinkages observed in our materials.

Supplementary Discussion 3: Calculation of theoretical shrinkage and density for the Fe_2O_3, Fe, CuO, Cu and Ag structures fabricated using our infusion-precipitation strategy.

<u>Fe_2O_3 and Fe</u>

From the TGA data shown in Figure S12, the Fe-ion infused 10-cycle Fe_3O_4 composites first experiences a 40% decrease in mass during the N_2 debinding step, followed by a 10% increase in mass in the following air sintering step. For ease of calculation, let us utilize the calculations made in Supplementary Discussion 2, i.e. the mass of an Fe-ion infused 10-cycle Fe_3O_4 composite is 127.14 g. We also assume that the Fe_3O_4 particles are completely converted to Fe_2O_3. If so, then the final mass of the Fe_2O_3 structure after the two-step debinding and sintering process is:

$$m_{IP-Fe_2O_3} = [(1 - 0.4) \times 127.14] \times 1.1 = 83.91\ g$$

The subscript *IP* refers to infusion-precipitation. If we assume the Fe_2O_3 structure was a cube, then the volume of the cube would be:

$$V_{IP-Fe_2O_3-theo} = \frac{83.91\ g}{\rho_{Fe_2O_3}} = \frac{83.91\ g}{5.24\ g/cm^3} = 16.01\ cm^3$$

The theoretical length of this Fe_2O_3 cube would thus be:

$$a_{IP-Fe_2O_3-theo} = \sqrt[3]{16.01} = 2.52\ cm.$$

From Supplementary Discussions 1 and 2, the mass of polymer (PEGda 700) in the dried Fe-ion infused 10-cycle Fe_3O_4 composite is 20 g. Assuming the density of PEGda 700 is 1.12 g/cm^3, the volume of 20 g of PEGda 700 is thus:

$$V_{PEGda700} = \frac{20\ g}{\rho_{PEGda\ 700}} = \frac{20\ g}{1.12} = 17.86\ cm^3$$

Since our hydrogels have a polymer to water volume ratio of 1:1, the volume of a hydrogel from 20 g of PEGda 700 is thus:

$$V_{IP-Hydrogel} = V_{PEGda700} + V_{H_2O} = 17.86 + 17.86 = 35.71\ cm^3$$

If we assume the hydrogel was a cube, with a volume of 35.71 cm^3, then the length of the hydrogel cube would be:

$$a_{IP-Hydrogel-theo} = \sqrt[3]{35.71} = 3.29\ cm.$$

Therefore, based on the mass loss data from the TGA plot, and the relative masses of the different components in the Fe-ion infused 10-cycle Fe_3O_4 composites, the theoretical linear shrinkage in going from the as-printed hydrogel to the Fe_2O_3 structure is:

$$Theoretical\ Shrinkage_{IP-Fe_2O_3} = \left(1 - \frac{a_{IP-Fe_2O_3-theo}}{a_{IP-Hydrogel-theo}}\right) \times 100\% = 23.46\%.$$

This theoretical linear shrinkage of 23.46% is consistent with our experimentally measured linear shrinkage of 20.47%.

To determine the theoretical density of our Fe_2O_3 structures, we compare the theoretical volume of the structure against the experimentally determined volume. We can estimate the experimentally determined volume by utilizing the experimentally determined linear shrinkage with our previously described hypothetical hydrogel cube. With a hydrogel-to-Fe_2O_3 experimental linear shrinkage of 20.47%, the length of a Fe_2O_3 cube from a hydrogel with length 3.29 cm is:

$$a_{IP-Fe_2O_3-exp} = (1 - 0.2047) \times 3.29 = 2.62\ cm$$

The volume of this Fe_2O_3 cube is thus:

$$V_{IP-Fe_2O_3-exp} = (2.62)^3 = 17.98\ cm^3$$

Thus, the theoretical densities of the Fe_2O_3 structures fabricated using our infusion-precipitation approach are:

$$Theoretical\ Density_{IP-Fe_2O_3} = \frac{V_{IP-Fe_2O_3-theo}}{V_{IP-Fe_2O_3-exp}} \times 100\ \% = \frac{16.01}{17.98} \times 100\ \% = 89.04\ \%.$$

To calculate the theoretical shrinkage for the Fe structures prepared via our infusion precipitation approach, we assume that the Fe_2O_3 structures described above are fully converted to Fe. The weight percent of Fe in Fe_2O_3 is given by:

$$wt\%_{Fe-in-Fe_2O_3} = \frac{2 \times 55.85\ g/mol}{159.69\ g/mol} \times 100\ \% = 69.95\ \%,$$

Thus, an Fe_2O_3 structure with a mass of 83.91 g (*vide supra*) will be converted to an Fe structure with a mass of 0.6995 x 83.91 g = 58.69 g.

If we assume the density of our Fe to be that of pure Fe, i.e. 7.87 g/cm^3, the volume of an Fe cube with a mass of 58.69 g will thus be 7.46 cm^3 ($V_{IP\text{-}Fe\text{-}theo}$). The length of a cube with this theoretical volume is thus:

$$\boldsymbol{a_{IP-Fe-theo} = \sqrt[3]{7.46} = 1.95\ cm}.$$

Therefore, the theoretical linear shrinkage in going from the as-printed hydrogel to the Fe structure is:

$$\boldsymbol{Theoretical\ Shrinkage_{IP-Fe} = \left(1 - \frac{a_{IP-Fe-theo}}{a_{IP-Hydrogel-theo}}\right) \times 100\% = 40.73\%}$$

This theoretically determined linear shrinkage is consistent with our experimentally determined value of 37.87%.

To determine the theoretical density of these Fe structures, we can follow the same procedure described above for the Fe_2O_3 structures. We can estimate the experimentally determined volume by utilizing the experimentally determined linear shrinkage with our previously described hypothetical hydrogel cube. With a hydrogel-to-Fe linear shrinkage of 37.87%, the length of a Fe cube from a hydrogel with length 3.29 cm is:

$$\boldsymbol{a_{IP-Fe-exp} = (1 - 0.3787) \times 3.29 = 2.04\ cm}$$

The volume of this Fe cube is thus:

$$\boldsymbol{V_{IP-Fe-exp} = (2.04)^3 = 8.49\ cm^3}$$

Thus, the theoretical densities of the Fe structures fabricated via the infusion-precipitation approach are:

$$\boldsymbol{Theoretical\ Density_{IP-Fe} = \frac{V_{IP-Fe-theo}}{V_{IP-Fe-exp}} \times 100\ \% = \frac{7.46}{8.49} \times 100\ \% = 87.9\%}.$$

Ag

From the TGA data shown in Figure S14, the 5-cycle Ag composites experience a total of 20.6% decrease in mass during the debinding and sintering process. For ease of calculation, let us assume that the starting 5-cycle Ag composite weighs 100 g. As such, the mass remaining after debinding and sintering is:

$$m_{IP-Ag} = (1 - 0.206) \times 100\ g = 79.4\ g$$

The subscript *IP* here denotes infusion-precipitation. We assume that the remaining mass is solely that of Ag. If we assume the Ag mass was a cube, then the volume of the cube would be:

$$V_{IP-Ag-theo} = \frac{79.4\ g}{\rho_{Ag}} = \frac{79.4\ g}{10.49\ g/cm^3} = 7.57\ cm^3$$

The theoretical length of this Ag cube would thus be:

$$a_{IP-Ag-theo} = \sqrt[3]{7.57} = 1.96\ cm$$

From Figure S8, the mass increase of the composite after 5 cycles of the infusion-precipitation process, as compared to the dried "blank" polymer was 400%. The mass increase was calculated according to the formula shown below:

$$mass\ increase = \frac{m_{5c} - m_{dbh}}{m_{dbh}} \times 100\ \%$$

Where m_{5c} is the mass of the dried 5-cycle composite and m_{dbh} is the mass of the dried "blank" hydrogel. For ease of calculation, let us assume that m_{5c} is 100 g and that the mass increase was entirely from the *in-situ* growth of Ag. Accordingly, the mass of polymer and Ag in the composite is thus 20 g and 80 g respectively. Assuming the density of PEGda 700 is 1.12 g/cm^3, the volume of 20 g of PEGda 700 is thus:

$$V_{PEGda700} = \frac{20\ g}{\rho_{PEGda\,700}} = \frac{20\ g}{1.12} = 17.86\ cm^3$$

Since our hydrogels have a polymer to water volume ratio of 1:1, the volume of a hydrogel from 20 g of PEGda 700 is thus:

$$V_{IP-Hydrogel} = V_{PEGda700} + V_{H_2O} = 17.86 + 17.86 = 35.71\ cm^3$$

If we assume the hydrogel was a cube, with a volume of 35.71 cm^3, then the length of the hydrogel cube would be:

$$\boldsymbol{a_{IP-Hydrogel-theo}} = \sqrt[3]{\mathbf{35.71}} = \mathbf{3.29}\ \boldsymbol{cm}.$$

The theoretical linear shrinkage in going from the as-printed hydrogel to the Ag structure can thus be determined by:

$$\boldsymbol{Theoretical\ Shrinkage_{Ag}} = \left(\mathbf{1} - \frac{\boldsymbol{a_{IP-Ag-theo}}}{\boldsymbol{a_{IP-Hydrogel-theo}}}\right) \times \mathbf{100\%} = \mathbf{40.42\%}$$

This theoretical linear shrinkage of 40.42% is consistent with our experimentally measured linear shrinkage of 36.67%.

To determine the theoretical density of our Ag structures, we compare the theoretical volume of the structure against the experimentally determined volume. We can estimate the experimentally determined volume by utilizing the experimentally determined linear shrinkage with our previously described hypothetical hydrogel cube. With a hydrogel-to-Ag linear shrinkage of 36.67%, the length of an Ag cube from a hydrogel with length 3.29 cm is:

$$\boldsymbol{a_{IP-Ag-exp}} = (\mathbf{1} - \mathbf{0.3667}) \times \mathbf{3.29} = \mathbf{2.084}\ \boldsymbol{cm}$$

The volume of this Ag cube is thus:

$$\boldsymbol{V_{IP-Ag-exp}} = (\mathbf{2.084})^{\mathbf{3}} = \mathbf{9.05}\ \boldsymbol{cm^3}$$

Thus, the theoretical densities of our Ag structures are:

$$\boldsymbol{Theoretical\ Density_{Ag}} = \frac{\boldsymbol{V_{IP-Ag-theo}}}{\boldsymbol{V_{IP\_Ag-exp}}} \times \mathbf{100}\ \% = \frac{\mathbf{7.57}}{\mathbf{9.05}} \times \mathbf{100}\ \% = \mathbf{83.60}\ \%.$$

Cu

From the TGA data shown in Figure S13, the 7-cycle Cu composites experience a 15% decrease in mass overall during the debinding and sintering process. For ease of calculation, let us assume that the starting 7-cycle Ag composite weighs 100 g. As such, the mass remaining after debinding and sintering is:

$$m_{IP-CuO} = (1 - 0.15) \times 100\ g = 85\ g$$

The subscript *IP* here denotes infusion-precipitation. We assume that the remaining mass is solely that of CuO. If we assume the CuO mass was a cube, then the volume of the cube would be:

$$V_{IP-CuO-theo} = \frac{85\ g}{\rho_{CuO}} = \frac{85\ g}{6.31\ g/cm^3} = 13.47\ cm^3$$

The theoretical length of this CuO cube would thus be:

$$a_{IP-CuO-theo} = \sqrt[3]{13.47} = 2.38\ cm$$

From Figure S9, the mass increase of the composite after 7 cycles of the infusion-precipitation process, as compared to the dried "blank" polymer was 210%. The mass increase was calculated according to the formula shown below:

$$mass\ increase = \frac{m_{7c} - m_{dbh}}{m_{dbh}} \times 100\ \%$$

Where m_{7c} is the mass of the dried 7-cycle composite and m_{dbh} is the mass of the dried "blank" hydrogel. For ease of calculation, let us assume that m_{7c} is 100 g and that the mass increase was entirely from the *in-situ* growth of Cu. Accordingly, the mass of polymer and Cu in the composite is thus 32 g and 68 g respectively. Assuming the density of PEGda 700 is 1.12 g/cm^3, the volume of 32 g of PEGda 700 is thus:

$$V_{PEGda700} = \frac{32\ g}{\rho_{PEGda\,700}} = \frac{32\ g}{1.12} = 28.57\ cm^3$$

Since our hydrogels have a polymer to water volume ratio of 1:1, the volume of a hydrogel from 32 g of PEGda 700 is thus:

$$V_{IP-Hydrogel} = V_{PEGda700} + V_{H_2O} = 28.57 + 28.57 = 57.14\ cm^3$$

If we assume the hydrogel was a cube, with a volume of 57.14 cm^3, then the length of the hydrogel cube would be:

$$a_{IP-Hydrogel-theo} = \sqrt[3]{57.14} = 3.85\ cm.$$

The theoretical linear shrinkage in going from the as-printed hydrogel to the CuO structure can thus be determined by:

$$Theoretical\ Shrinkage_{CuO} = \left(1 - \frac{a_{IP-CuO-theo}}{a_{IP-Hydrogel-theo}}\right) \times 100\% = 38.18\%$$

This theoretical linear shrinkage of 38.18% is in the same order as our experimentally measured linear shrinkage of 30.60%.

To determine the theoretical density of our CuO structures, we compare the theoretical volume of the structure against the experimentally determined volume. We can estimate the experimentally determined volume by utilizing the experimentally determined linear shrinkage with our previously described hypothetical hydrogel cube. With a hydrogel-to-CuO experimental linear shrinkage of 30.60%, the length of a CuO cube from a hydrogel with length 3.85 cm is:

$$a_{IP-CuO-exp} = (1 - 0.3060) \times 3.85 = 2.67\ cm$$

The volume of this CuO cube is thus:

$$V_{IP-CuO-exp} = (2.67)^3 = 19.03\ cm^3$$

Thus, the theoretical densities of the CuO structures fabricated using our infusion-precipitation approach are:

$$Theoretical\ Density_{IP-CuO} = \frac{V_{IP-CuO-theo}}{V_{IP-CuO-exp}} \times 100\ \% = \frac{13.47}{19.03} \times 100\ \% = 70.78\ \%.$$

To calculate the theoretical shrinkage for the Cu structures prepared via our infusion precipitation approach, we assume that the CuO structures described above are fully converted to Cu. The weight percent of Cu in CuO is given by:

$$\boldsymbol{wt\%_{Cu-in-CuO} = \frac{63.55\ g/mol}{79.55\ g/mol} \times 100\ \% = 79.89\ \%},$$

Thus, an CuO structure with a mass of 85 g (*vide supra*) will be converted to an Cu structure with a mass of 0.7989 x 85 g = 67.91 g.

If we assume the density of our Cu to be that of pure Cu, i.e. 8.96 g/cm^3, the volume of an Cu cube with a mass of 67.91 g will thus be 7.58 cm^3 ($V_{IP\text{-}Cu\text{-}theo}$). The length of a cube with this theoretical volume is thus:

$$\boldsymbol{a_{IP-Cu-theo} = \sqrt[3]{7.58} = 1.96\ cm}.$$

Therefore, the theoretical linear shrinkage in going from the as-printed hydrogel to the Fe structure is:

$$\boldsymbol{Theoretical\ Shrinkage_{IP-Fe} = \left(1 - \frac{a_{IP-Cu-theo}}{a_{IP-Hydrogel-theo}}\right) \times 100\% = 49.09\%}$$

This theoretically determined linear shrinkage is consistent with our experimentally determined value of 46.00%.

To determine the theoretical density of these Cu structures, we can follow the same procedure described above for the CuO structures. We can estimate the experimentally determined volume by utilizing the experimentally determined linear shrinkage with our previously described hypothetical hydrogel cube. With a hydrogel-to-Cu linear shrinkage of 46.00%, the length of a Cu cube from a hydrogel with length 3.85 cm is:

$$\boldsymbol{a_{IP-Cu-exp} = (1 - 0.4600) \times 3.85 = 2.08\ cm}$$

The volume of this Cu cube is thus:

$$\boldsymbol{V_{IP-Cu-exp} = (2.08)^3 = 9.00\ cm^3}$$

Thus, the theoretical densities of the Cu structures fabricated via the infusion-precipitation approach are:

$$\boldsymbol{Theoretical\ Density_{IP-Cu}} = \frac{\boldsymbol{V_{IP-Cu-theo}}}{\boldsymbol{V_{IP-Cu-exp}}} \times \mathbf{100}\ \% = \frac{\mathbf{7.58}}{\mathbf{9.00}} \times \mathbf{100}\ \% = \mathbf{84.22}\%.$$

Supplementary Discussion 4: Determining the experimental density of the metal structures using μCT

In this study, we elected to use a μCT-based density measurement method to experimentally determine the densities of our printed metal structures. [16] We considered the Archimedes method, which is a typical method to measure average volumetric density of AM metal parts, however, there are problems with using it for measuring the small structures fabricated in this study. For example, air bubbles can attach to the small channels of the structure due to surface tension, resulting in larger volume measurement and hence lower measured density. We further considered using mercury intrusion porosimetry (MIP), but the high injection pressures needed risked damaging the sample and skewing the porosity measurements.

μCT-based density measurement method:

In brief, we first measured the mass of the metal gyroids, $\boldsymbol{m_{experimental}}$, with a weighing balance. The volume of each sample, $\boldsymbol{V_{experimental}}$, was then obtained by using μCT reconstruction and CloudCompare (open-source software) calculation. The experimental densities of the samples were then calculated using:

$$\boldsymbol{Experimental\ density} = \frac{\boldsymbol{m_{experimental}}}{\boldsymbol{V_{experimental}}} / \boldsymbol{\rho_{metal}}$$

where $\boldsymbol{\rho_{metal}}$ is the density of the metal.

As shown in Table S3 below, the experimental densities of the Fe and Cu structures were ~ 3 percentage points higher than their theoretical densities, while the experimental density of the Ag structures were ~ 7 percentage points lower.

Table S3. Experimental and theoretical density of metals made using HIAM and our infusion-precipitation method.

Material	Experimental density (%)	Theoretical density (%)
Fe (HIAM)	50	49
Fe	91	88
Cu	88	84
Ag	76	84

We attribute the lower experimental density of the Ag structures to the lower quality of their μCT images. As mentioned in the main text, Ag is generally difficult to image with X-ray μCT due to its strong X-ray attenuation properties. This causes severe artefacts and blurred boundaries, which results in volume reconstructions with poor qualities (Figure S33a). We attempted to use an X-ray source with a Cu filter to try and improve the μCT imaging quality, but found that although there were less artefacts, the sliced images still exhibited blurred boundaries (Figure S33b). These blurred boundaries introduced inaccuracies when segmenting the sample from the background, i.e. the background is recognized as the sample during reconstruction of the μCT scan. As a result, the reconstructed Ag structures exhibit artificially larger volumes than the actual Ag samples, which in turn lead to underestimated density values.

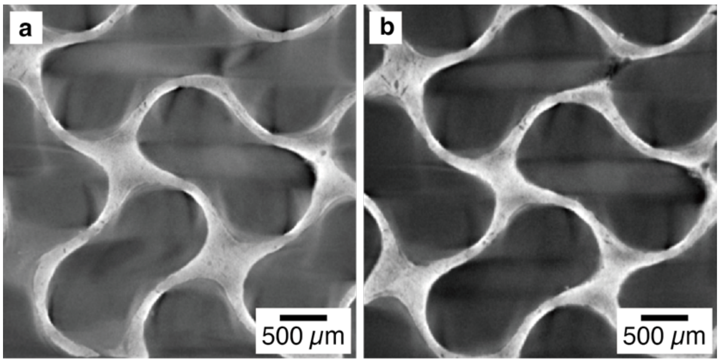

Figure S33. X-ray μCT scan of a silver gyroid a) without Cu filter and b) with Cu filter. Although the Cu filter helped to remove the scanning artefacts, the sliced images still exhibited blurred boundaries.

It is important to note that the μCT scans of the Fe and Cu structures do not have such blurred boundaries (Figure S19 and S20), i.e. the reconstructed volumes are close to that of the true volume of the structure. Since the experimental densities of the Fe and Cu structures are relatively consistent with their theoretical densities, we are confident that the theoretical densities of the Ag structures are similarly close to their true values. The experimentally determined densities of the Ag are, as explained above, likely to be artificially lowered due to the challenges with scanning them in the μCT.

Taken together, we expect that our theoretical density calculation method is representative of the true densities of the structures and can thus be broadly applied to metals fabricated via the metal-salt VP approach. Furthermore, for most metals produced with these methods, the μCT-based density measurement method is an effective method for experimentally measuring their densities.

Supplementary Discussion 5: Calculation of theoretical density from other relevant metal-salt solution VP studies

In this Supplementary Discussion, we describe the linear shrinkages and theoretical densities of materials in other relevant metal-salt solution VP studies. If the theoretical densities of the fabricated materials are not described in the study, we detail how we calculate them. We also explain why the calculation of the theoretical densities are sometimes not possible from the information in the study.

Relevant Work 1: Saccone et al. *Nature* 2022, 612, 7941, 685-690 (HIAM)

This work is also referred to as HIAM in the main text. Since a variety of materials were fabricated, we selected Cu and Ag as representative materials.

Cu

The reported experimental linear shrinkage from as-printed to metal of the HIAM Cu was 63.04 % ($Shrinkage_{HIAM\text{-}Cu\text{-}exp}$). The reported mass loss from their ion-infused hydrogel to ceramic was 87.32 % ($\Delta m_{HIAM\text{-}CuO}$). Let us first calculate the theoretical shrinkage. For ease of calculation, let us assume that the mass of the Cu-ion infused hydrogel is 100 g, the mass of CuO after thermal treatment is thus:

$$m_{HIAM-CuO} = (1 - 0.8732) \times 100g = 12.68\,g$$

The theoretical mass of Cu after reducing in forming gas would thus be:

$$m_{HIAM-Cu} = \frac{12.68\,g}{M_{CuO}} \times M_{Cu} = \frac{12.68\,g}{79.55\,g/mol} \times 63.55\frac{g}{mol} = 10.13\,g$$

Where M_{CuO} and M_{Cu} are the molar masses of CuO and Cu respectively. If we assume the Cu structure was a cube, then the volume of the cube would be:

$$V_{HIAM-Cu-theo} = \frac{10.31\,g}{\rho_{Cu}} = \frac{10.31\,g}{8.96\,g/cm^3} = 1.15\,cm^3$$

The theoretical length of this Cu cube would thus be:

$$a_{HIAM-Cu-theo} = \sqrt[3]{1.15} = 1.05\,cm.$$

To calculate the theoretical volume of the as-printed hydrogel, we first need to determine its mass. From the mass of CuO left after calcination, the mass of $Cu(NO_3)_2{\cdot}6H_2O$ that was infused into the 100 g Cu-ion infused hydrogel can be estimated as such:

$$m_{HIAM-Cu\_salt} = \frac{m_{HIAM-CuO}}{M_{CuO}} \times M_{Cu(NO_3)_2\cdot 6H_2O} = \frac{12.68\ g}{79.55\ g/mol} \times 297.49\ g/mol = 47.42\ g.$$

Where $M_{Cu(NO3)2\cdot 6H2O}$ is the molar mass of $Cu(NO_3)_2{\cdot}6H_2O$. The amount of water in the Cu-ion infused hydrogel, as estimated from their TGA measurements (Figure 3 in the HIAM paper), was taken to be 20 g (20 wt% loss; assuming the Cu-ion infused hydrogel was 100 g). Since PEGda 575 was used as the polymer in this study, the mass of PEGda 575 in the dried Cu-ion infused hydrogel ($m_{HIAM\text{-}PEGda\ 575}$) can thus be given by:

$$m_{HIAM-PEGda\,575} = 100\ g - 20g - m_{HIAM-Cu\_salt} = 32.58\ g$$

Using the density of PEGda 575 as 1.12 g/cm^3, this corresponds to a volume of 29.08 cm^3 ($V_{PEGda\ 575}$) As reported in the methods section of the paper, the volume ratio between DMF and PEGda 575 was 1:1. Thus, the volume of the organogels as printed can be given by:

$$V_{HIAM-Organogel} = V_{PEGda\,575} + V_{DMF} = 29.08 + 29.08 = 58.16\ cm^3$$

If we assume that the organogels are in the shape of a cube, then the theoretical length of this organogel cube is:

$$a_{HIAM-Organogel-theo} = \sqrt[3]{V_{HIAM-Organogel}} = 3.87\ cm$$

With the experimentally measured linear shrinkage of 63.04 %, the length of a Cu cube from this organogel cube would thus be:

$$a_{HIAM-Cu-exp} = (1 - 0.634) \times 3.87 = 1.42\ cm$$

The theoretical density of the Cu cube structure can thus be given by:

$$Theoretical\ Density_{HIAM-Cu} = \frac{V_{HIAM-Cu-theo}}{V_{HIAM-Cu-exp}} \times 100\ \% = \frac{(1.05)^3}{(1.42)^3} \times 100\ \% = 40.4\%$$

Ag:

The reported experimental linear shrinkage from as-printed to metal of the HIAM Ag was 57.35 % ($Shrinkage_{HIAM\text{-}Ag\text{-}exp}$). The reported mass loss from their ion-infused hydrogel to metal was 66.13 % ($\Delta m_{HIAM\text{-}Ag}$). Let us first calculate the theoretical shrinkage. For ease of calculation, let us assume that the mass of the Ag-ion infused hydrogel is 100 g, the mass of Ag after thermal treatment is thus:

$$m_{HIAM-Ag} = (1 - 0.6613) \times 100g = 33.87\ g$$

If we assume the Ag structure was a cube, then the volume of the cube would be:

$$V_{HIAM-Ag-theo} = \frac{33.87\ g}{\rho_{Ag}} = \frac{33.87\ g}{10.5\ g/cm^3} = 3.23\ cm^3$$

The theoretical length of this Ag cube would thus be:

$$a_{HIAM-Ag-theo} = \sqrt[3]{3.23} = 1.48\ cm.$$

To calculate the theoretical volume of the as-printed hydrogel, we first need to determine its mass. From the mass of Ag left after calcination, the mass of $AgNO_3$ that was infused into the 100 g Ag-ion infused hydrogel can be estimated as such:

$$m_{HIAM-Ag\_salt} = \frac{m_{HIAM-Ag}}{M_{Ag}} \times M_{AgNO_3} = \frac{33.87\ g}{107.87\ g/mol} \times 169.87 \frac{g}{mol} = 53.34\ g$$

Where M_{AgNO3} is the molar mass of $AgNO_3$. The amount of water in the Ag-ion infused hydrogel, as estimated from their TGA measurements (Figure S10 in the HIAM paper), was taken to be 2 g (2 wt% loss; assuming the Ag-ion infused hydrogel was 100 g). Since PEGda 575 was used as the polymer in this study, the mass of PEGda 575 in the dried Ag-ion infused hydrogel ($m_{HIAM\text{-}PEGda\ 575}$) can thus be given by:

$$m_{HIAM-PEGda\,575-Ag} = 100\ g - 2g - m_{HIAM-Ag_{salt}} = 44.66\ g$$

Using the density of PEGda 575 as 1.12 g/cm^3, this corresponds to a volume of 39.88 cm^3 ($V_{PEGda\ 575\text{-}Ag}$) As reported in the methods section of the paper, the volume ratio between DMF and PEGda 575 was 1:1. Thus, the volume of the organogels as printed can be given by:

$$V_{HIAM-Organogel-Ag} = V_{PEGda\,575-Ag} + V_{DMF-Ag} = 39.88 + 39.88 = 79.76\ cm^3$$

If we assume that the organogels are in the shape of a cube, then the theoretical length of this organogel cube is:

$$a_{HIAM-Organogel-Ag-theo} = \sqrt[3]{V_{HIAM-Organogel-Ag}} = 4.30\ cm$$

With the experimentally measured linear shrinkage of 57.35 %, the length of a Cu cube from this organogel cube would thus be:

$$a_{HIAM-Ag-exp} = (1 - 0.5735) \times 4.30 = 1.83\ cm$$

The theoretical density of the Ag cube structure can thus be given by:

$$Theoretical\ Density_{HIAM-Ag} = \frac{V_{HIAM-Ag-theo}}{V_{HIAM-Ag-exp}} \times 100\ \% = \frac{(1.48)^3}{(1.83)^3} \times 100\ \% = 52.90\%$$

Relevant Work 2: Martinez et al. *Adv. Manuf,* 2024, DOI: 10.1007/s40436-024-00514-z (HIAM)

Martinez et al. report the fabrication of Cu structures using a similar approach to that outlined by Saccone et al. They report a linear shrinkage of 65 % and a mass loss of 85.5 %. These values are similar to that reported by Saccone et al. Using the same logic described above, and with a water content of about 10 wt% (from their TGA data in Figure 2), we can determine the following for a 100 g Cu-ion infused hydrogel:

$$a_{Martinez-Cu-theo} = 1.09\ cm$$

$$a_{Martinez-Organogel-theo} = 4.00\ cm$$

$$a_{Martinez-Cu-exp} = 1.40\ cm$$

$$Theoretical\ Density_{Martinez-Cu} = \frac{(1.09)^3}{(1.40)^3} \times 100\ \% = 47.2\%$$

Relevant Work 3: Saigal et al. *Proceedings of the ASME 2023 Aerospace Structures, Structural Dynamics, and Materials Conference. ASME 2023 Aerospace Structures, Structural Dynamics, and Materials Conference*. San Diego, California, USA. June 19–21, 2023. V001T03A007. ASME. https://doi.org/10.1115/SSDM2023-107356 (HIAM)

Saigal et al. demonstrate the fabrication of Ag using a HIAM process. They report a volumetric reduction of 57.4 %. However, in measuring the dimensions of their springs shown in Figures 6 and 8, we assume that this was a typographic error and that the authors meant to say that the experimentally measured linear shrinkage was 57.4 %. Their TGA data indicates that a mass loss of 85% occurred during their process. Assuming a hydrated Ag-ion infused hydrogel of 100 g, this means that a final mass of 15 g of Ag was obtained. Taking the density of Ag to be 10.49 g/cm^3, the volume of this Ag is thus 1.43 cm^3. Assuming a cube of this volume, the length, a, of this Ag cube can thus be determined by:

$$\boldsymbol{a_{Saigal-Ag-theo} = \sqrt[3]{1.43} = 1.13\ cm}$$

From the mass of Ag left in the system, we can estimate the mass of $AgNO_3$ that was in the hydrogel:

$$\boldsymbol{m_{AgNO_3} = \frac{15\ g}{M_{Ag}} \times M_{AgNO_3} = \frac{15\ g}{107.87\ g/mol} \times 169.87\frac{g}{mol} = 23.62\ g}$$

Since the TGA was conducted on a hydrated hydrogel, we need to take the amount of water in the hydrogel into consideration. From the TGA, the mass of water in the hydrated hydrogel can be estimated to be ~ 27 wt%, i.e. 27 g in our 100 g example. Thus, the mass of polymer (PEGda 575) in their hydrated Ag-ion infused hydrogel is 49.38 g. According to their resin formulation Table S1, their resin contains 37.4 and 61.8 wt% of dimethylformamide (DMF) and PEGda 575 respectively. For ease of calculation, we will include the wt% of the photosensitive reagents (< 1 wt%) into the DMF, i.e. the resin contains 38.2 wt% of DMF. Using a density of 0.944 g/cm^3 and 1.12 g/cm^3 for DMF and PEGda 575 respectively, and assuming a total resin mass of 100

g, the volume of DMF and PEGda 575 in this resin can be determined to be 40.47 and 55.18 cm^3 respectively. The volume fractions of DMF and PEGda 575 in the resin are thus 42.3 and 57.7 % respectively. Given that, we assume that the as-printed organogels have a DMF volume percentage of 42.3 %. We also assume that on solvent exchange, all the DMF is exchanged with water, i.e. the hydrogel also has a volume percentage of 42.3 %. As described above, the mass of PEGda 575 in our hypothetical 100 g of hydrated Ag-ion infused hydrogel was theoretically determined to be 49.38 g. This translates to a volume of 44.09 cm^3. Accordingly, the "blank" hydrogel should thus have 32.32 cm^3 of water (from (44.09 / 57.7) x 42.3). The "blank" hydrogel thus has a volume of 76.41 cm^3. Assuming a cube of this volume, the length, a, of the initial "blank" hydrogel cube can thus be determined by:

$$a_{Saigal-hydrogel-theo} = \sqrt[3]{76.41} = 4.24\ cm$$

From their experimentally measured linear shrinkage, this "blank" hydrogel cube should thus shrink linearly by 57.4 % to yield a cube with length:

$$a_{Saigal-Ag-exp} = (1 - 0.574) \times 4.24 = 1.81\ cm$$

The theoretical density of the Ag cube structure can thus be given by:

$$Theoretical\ Density_{Saigal-Ag} = \frac{V_{Saigal-Ag-theo}}{V_{Saigal-Ag-exp}} \times 100\ \% = \frac{(1.13)^3}{(1.81)^3} \times 100\ \% = 24.3\%$$

Relevant Work 4: Ma et al. *Angew. Chem., Int. Ed.* 2024, 63, 23, e202405135 (HIAM)

Ma and colleagues demonstrate the fabrication of Cu using a HIAM process. They reported a theoretical linear shrinkage of 66.8 % and an experimentally measured linear shrinkage of 60 %. Their theoretical density is thus:

$$Theoretical\ Density_{Ma-Cu-theo} = \frac{(1 - 0.668)^3}{(1 - 0.600)^3} \times 100\ \% = 57.18\ \%$$

Relevant Work 5: Sun et al. *Adv. Funct. Mater.* 2024, 2418035 (HIAM)

Sun et al. demonstrated the fabrication of iron foams using an iron-ion infusion process. They reported a linear shrinkage of approximately 55 %, with an associated mass loss of approximately 65 %. However, since their work revolved around the fabrication of structures with multi-scale porosity, the determination of theoretical density is not applicable here.

Relevant Work 6: Zhang et al. *Nano Lett. 2023*, 23, 17, 8162-8170 (HIAM)

Zhang et al. demonstrated the fabrication of nickel pillars using a HIAM process. They report that their printed pillars (diameter 2 µm) shrank to a diameter between 130 – 550 nm. This translates to a linear shrinkage between 72.5 – 93.5 %. They report a porosity between 10 – 25 %, which indicates a density between 75 – 90 %. The average linear shrinkage value of 83% and the average density value of 82.5% were used for Figure 6.

Relevant Work 7: Yee et al. *Adv. Mater.* 2019, 31, 33, 1901345

Yee et al. demonstrated the fabrication of zinc oxide structures using a hydrogel that contained zinc nitrate. They report the fabrication of ZnO structures with experimentally measured linear shrinkages between 70 – 90%. The theoretical density cannot be determined since the mass of zinc nitrate in the hydrogel is a function of its post-printing development time and thus, cannot be measured accurately. The reported mass loss in their TGA data is from a bulk sample that was not developed in water, i.e. this TGA data is not applicable to the 3D printed samples.

Relevant Work 8: Yee et al. *Adv. Mater. Technol.* 2021, 6, 2, 2000791

Yee et al. demonstrated the fabrication of lithium cobalt oxide structures using a hydrogel that contained lithium nitrate and cobalt nitrate. They report an experimentally measured linear shrinkage of 44 % and a theoretical density of 44 %.

Relevant Work 9: Cai et al. *Adv. Sci.* 2024, 11, 39, 2405487

Cai et al. demonstrated the fabrication of tungsten structures using a hydrogel that contained ammonium metatungstate. In their work, they report a linear shrinkage factor of 40 % and a theoretical linear shrinkage factor of 38.5 %. However, linear shrinkage factor, as defined by the authors, is the length of the feature remaining. In our calculations, we define linear shrinkage as the length that has been reduced. Hence, using our terminology, in this work, the measured linear shrinkage is 60 % and the theoretical linear shrinkage is 61.5 %. Their theoretical density is thus:

$$\boldsymbol{Theoretical\ Density_{Cai-W-theo}} = \frac{(\mathbf{1-0.615})^{3}}{(\mathbf{1-0.6})^{3}} \times \mathbf{100}\ \% = \mathbf{89.1}\ \%$$

Relevant Work 10: Xiong et al. *Adv. Mater.* 2024, 36, 32, 2405053

Xiong et al. report the fabrication of a variety of metal oxides using hydrogels that contain metal salts. They report linear shrinkages between 30 – 55 % for their metal oxides. The theoretical density was not calculated since the relevant data was not available.

Relevant Work 11: Rosental et al. *Chem. Eng. J.* 2024, 499, 156189

Rosental et al. fabricated barium titanate structures using polymers that contained barium acetate and titanium alkoxide. It is worth noting that this work is not strictly a metal-salt solution method since it also uses a sol-gel process. It is included here for completeness. The authors report a linear shrinkage of 60 % and a density of 98 %.

Relevant Work 12: Douvdevany et al. *J. Eur. Ceram. Soc.* 2024, 44, 15, 116773.

Douvdevany et al. fabricated ruby (Cr doped Al_2O_3) using organogels that contained aluminium chloride and chromium chloride. Similar to work 9, this work is also a sol-gel process. They

report a linear shrinkage of 72% and a theoretical density between 75 – 90% (based on polycrystalline alumina). The average value of 83% was used for Figure 6.

Relevant Work 13: Zanini et al. *Adv. Funct. Mater.* 2024, 34, 46, 2406916

Zanini et al. fabricated uranium dicarbide structures using polymers that contained uranyl nitrate as the metal precursor. This work also utilized a sol-gel process. They report an experimental linear shrinkage of 50 – 55 % and a theoretical shrinkage of 64.4 %. Using their experimental shrinkage of 55 %, their theoretical density can be calculated as:

$$\boldsymbol{Theoretical\ Density_{Zinini-UC-theo}} = \frac{(\mathbf{1-0.644})^3}{(\mathbf{1-0.55})^3} \times \mathbf{100}\ \% = \ \mathbf{49.5}\ \%$$

Relevant Work 14: Luitz et al. *Adv. Eng. Mater.* 2023, 25, 13, 2201927

Luitz et al. fabricated tungsten structures using hydrogels that contained sodium metatungstate. They report an experimental linear shrinkage of 53.4 % and a density of 54.1 % (based on their reported porosity of 45.9 %).

Relevant Work 15: Zan et al. *J. Phys. D Appl. Phys.* 2022, 55, 444004

Zan et al. fabricated tungsten structures using hydrogels that contained ammonium metatungstate. The experimental linear shrinkage as measured from the heights of their Rook images in Figure 1c-1 and Figure 1c-3 was 41.2 %. Their measured density ranged from 64 – 83%. The average value of 74% was used for Figure S21.

Supplementary Discussion 6: Calculation of theoretical density of HIAM Fe_2O_3 and Fe

From the HIAM Fe_2O_3TGA data, we can determine that there is a mass decrease of 84 % from the thermal conversion of the Fe-ion infused hydrogel to the Fe_2O_3 structure. For ease of calculation, let us assume the mass of the Fe-ion infused hydrogel is 100 g. The mass of the remaining Fe_2O_3 ($m_{HIAM\text{-}Fe2O3}$) is thus 16 g. Taking the density of Fe_2O_3 to be 5.24 g/cm^3, the volume of this mass is thus 3.05 cm^3. If we assume the Fe_2O_3 structure was a cube, then the theoretical length of this Fe_2O_3 cube would thus be:

$$a_{HIAM-Fe_2O_3-theo} = \sqrt[3]{3.05} = 1.45\ cm.$$

The number of Fe-ions in the system can thus be determined by:

$$n_{HIAM-Fe-ion-hydrogel} = \frac{m_{HIAM-Fe_2O_3}}{159.69\ g/mol} \times 2 = 0.20\ mol$$

If we assume that the ratio of Fe^{2+} and Fe^{3+} ions in the Fe-ion infused hydrogel is the same as the infusion solution, then the ratio of Fe^{2+}: Fe^{3+} should be 1:1.8. Accordingly, the mass of iron salts in the Fe-ion infused hydrogel can be determined by:

$$m_{HIAM-FeCl_2\cdot 4H_2O} = \frac{n_{HIAM-Fe-ion-hydrogel}}{2.8} \times 1.0 \times M_{FeCl_2\cdot 4H_2O} = 14.21\ g$$

$$m_{HIAM-FeCl_3\cdot 6H_2O} = \frac{n_{HIAM-Fe-ion-hydrogel}}{2.8} \times 1.8 \times M_{FeCl_3\cdot 6H_2O} = 34.76\ g$$

From the TGA data in Figure S11, the Fe-ion infused hydrogel had approximately 10 wt% of water i.e. a water mass of 10 g. The mass of polymer in 100 g of Fe-ion infused hydrogel ($m_{HIAM\text{-}polymer}$) is thus 100 – 10 – 14.21 – 34.76 = 41.03 g. Assuming the density of PEGda 700 is 1.12 g/cm^3, the volume of this polymer mass is thus 36.63 cm^3. Since the ratio of water to PEGda 700 is 1:1, the volume of the hydrogel before infusion can thus be estimated to be:

$$V_{HIAM-Hydrogel} = V_{PEGda700} + V_{H_2O} = 36.63 + 36.63 = 73.26\ cm^3$$

If we assume the hydrogel was a cube, with a volume of 73.26 cm^3, then the length of the hydrogel cube would be:

$$a_{HIAM-Hydrogel-theo} = \sqrt[3]{73.26} = 4.18\ cm.$$

Thus, the theoretical linear shrinkage in going from the as-printed hydrogel to the Fe_2O_3 structure in the HIAM process is:

$$\boldsymbol{Theoretical\ Shrinkage_{HIAM-Fe_2O_3}} = \left(\mathbf{1} - \frac{a_{HIAM-Fe_2O_3-theo}}{a_{HIAM-Hydrogel-theo}}\right) \times \mathbf{100\%} = \mathbf{65.31\%}.$$

Our experimentally measured linear shrinkage for HIAM Fe_2O_3 is 42.9 %. Accordingly, we can calculate the theoretical density to be:

$$\boldsymbol{Theoretical\ Density_{HIAM-Fe_2O_3-theo}} = \frac{(\mathbf{1-0.6531})^3}{(\mathbf{1-0.4290})^3} \times \mathbf{100}\ \% = \mathbf{22.4}\ \%$$

If we assume the complete reduction from Fe_2O_3 to Fe, the 16 g of Fe_2O_3 is converted to 11.17 g of Fe. Taking the density of Fe to be 7.87 g/cm^3, this mass of Fe has a volume of 1.42 cm^3. If we assume the Fe was a cube, with a volume of 1.42 cm^3, then the length of the Fe cube would be:

$$\boldsymbol{a_{HIAM-Fe-theo}} = \sqrt[3]{\mathbf{1.42}} = \mathbf{1.12}\ \boldsymbol{cm}.$$

Therefore, the theoretical shrinkage for HIAM Fe is

$$\boldsymbol{Theoretical\ Shrinkage_{HIAM-Fe}} = \left(\mathbf{1} - \frac{a_{HIAM-Fe-theo}}{a_{HIAM-Hydrogel-theo}}\right) \times \mathbf{100}\ \% = \mathbf{73.20}\ \%$$

Our experimentally measured linear shrinkage as-printed hydrogel to Fe was 65.93%. Accordingly, we can calculate the theoretical density of the HIAM Fe to be:

$$\boldsymbol{Theoretical\ Density_{HIAM-Fe-theo}} = \frac{(\mathbf{1-0.7320})^3}{(\mathbf{1-0.6593})^3} \times \mathbf{100}\ \% = \mathbf{48.7}\ \%$$

Supplementary Discussion 7: Nanoindentation of Fe, Cu, and Ag structures fabricated via the infusion-precipitation method.

We conducted additional nanoindentation experiments on our Fe, Cu, and Ag metal structures to better understand their properties. The metal gyroids were fabricated, mounted in epoxy, polished, and then indented with a Berkovich tip. The results are summarized in Table S4 below.

Table S4. Elastic modulus and hardness of metals made using our infusion-precipitation method.

Material	Hardness (Hv)	Elastic Modulus (GPa)
Fe	178 ± 19	81 ± 6
Cu	127 ± 16	53 ± 3
Ag	123 ± 2	34 ± 1

The elastic moduli of our metals are consistently lower than that of their bulk counterparts. We attribute this to the presence of porosity in our metal structures, which ranges from 12 – 16 vol% depending on the specific metal. This level of porosity, while relatively low compared to the state of the art, still significantly affects the stiffness of the material. Choren et al. showed that at a porosity of 15 vol%, the expected elastic modulus is 50 – 83% of that of a fully dense material, depending on the analytical model used. [17] Considering the porosity levels in our metal structures (10 – 20 vol%), our measured elastic moduli values fall well within these predicted ranges. The work by Choren et al. also highlights the importance of increasing material density since even a porosity of 5% can lead to a decrease in modulus by 25%.

The hardness values of our metals exceed the range typically expected from their bulk counterparts. Similar increases in hardness have been reported previously by both Saccone et al. [1] and Ma et al. [4] in their respective HIAM-based studies. The underlying cause of this increased hardness remains unclear and is beyond the scope of the present work. One possible factor is the presence of carbon, which cannot be accurately quantified in our study. Residual carbon could result in the formation of new phases or alloys that harden the material.

Supplementary Discussion 8: Dimensional fidelity as a function of structure size

The reduced dimensional fidelity of the smaller Ag gyroid in Figure 5d can be attributed to the intrinsic resolution limits of the DLP printer rather than to the infusion–precipitation chemistry itself. The printer used in this work was a MONO3-MZ4 (MonoPrinter) with a lateral (XY) resolution of 27 µm and a vertical (Z) resolution set to 50 µm. During the slicing process, i.e., the conversion of CAD files into print code, jagged artifacts inevitably arise along smooth curves, manifesting as pixelation in the XY plane and layered patterns in the Z direction (Figure S34). The layered patterns are present in all DLP-printed hydrogel structures, which are then retained in the metal structures. This can be clearly observed in Figure 3c, f, and i.

Small-scale structures are disproportionately affected by these jagged artifacts and also have increased printed inaccuracies due to the relative size of their feature to the pixel size. For example, the gyroid templates used to fabricate the small mm-scale Ag structures had a target wall thickness of 45 µm, which, depending on the curvature of the CAD file, corresponded to 2–3 pixels. As such, these meant that the wall had a thickness of either 54 µm (2 pixels) or 81 µm (3 pixels), which is an error of up to ~80% compared to the CAD file. In contrast, the larger gyroids used to fabricate the silver structures in Figure 3a had wall thicknesses that were approximately three times thicker (~150 µm, equivalent to 5–6 pixels) and exhibited far smaller deviations, typically within ~10%. This illustrates that the pixelation effect disproportionately compromise the fidelity of small structures, while their impact on larger features is comparatively minor. We expect that the use of a printer with smaller pixel sizes will reduce this effect.

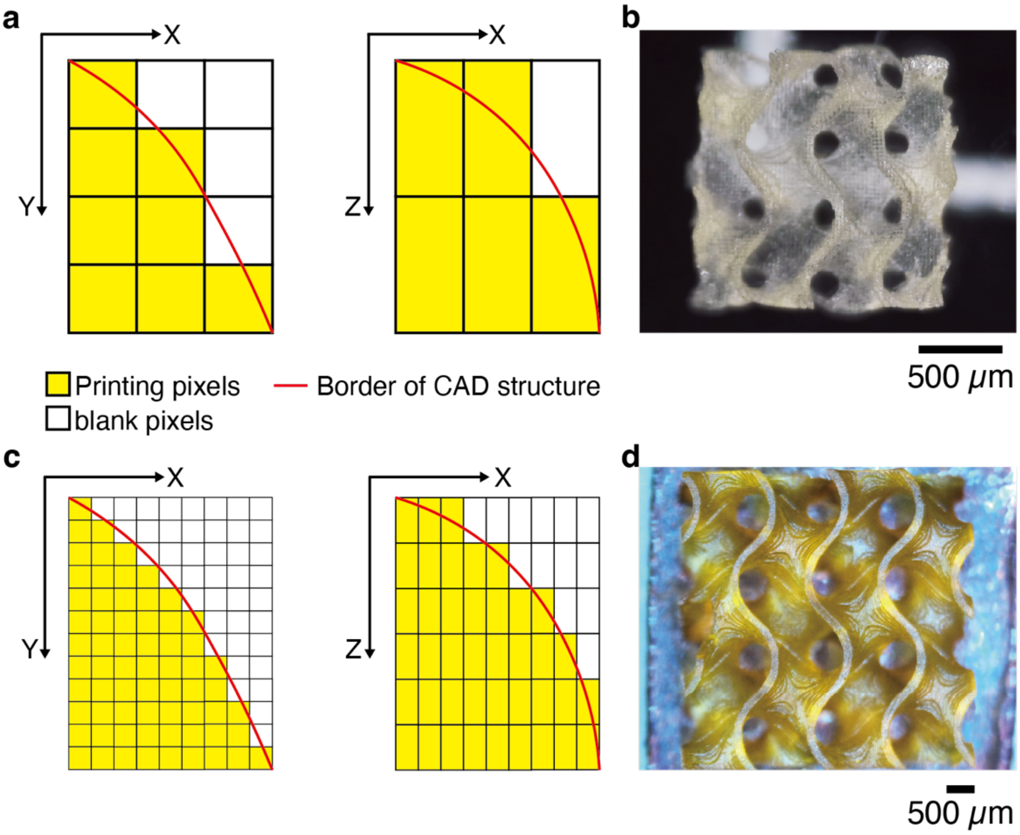

Figure S34. a,c) Schematics illustrating that the impact of the jagged artifacts decreases with the number of pixels used to print the feature. Note that the pixel sizes in (a) and (c) are identical. The size of the polymer template used to print the silver structures in Figure 5d and Figure 3a are shown in b) and d) respectively. Since b) is significantly smaller than d), we expect the jagged artifacts in b) to be more pronounced than in d), which would contribute to the deviation from the CAD file.

References

[1] Saccone et al., *Nature* **2022**, *612*, 685.
[2] Martinez et al., *Adv. Manuf.* **2024**, DOI 10.1007/s40436-024-00514-z.
[3] Saigal et al., in *ASME 2023 Aerospace Structures, Structural Dynamics, and Materials Conference*, American Society Of Mechanical Engineers, San Diego, California, USA, **2023**, p. V001T03A007.
[4] Ma et al., *Angew Chem Int Ed* **2024**, *63*, e202405135.
[5] Sun et al., *Adv Funct Materials* **2024**, 2418035.
[6] Zhang et al., *Nano Lett.* **2023**, acs.nanolett.3c02309.
[7] Yee et al., *Adv. Mater.* **2019**, *31*, 1901345.
[8] Yee et al., *Adv Materials Technologies* **2021**, *6*, 2000791.
[9] Cai et al., *Advanced Science* **2024**, 2405487.
[10] Hu et al., *Advanced Materials* **2024**, *36*, 2405053.
[11] Rosental et al., *Chemical Engineering Journal* **2024**, *499*, 156189.
[12] Moshkovitz Douvdevany et al., *Journal of the European Ceramic Society* **2024**, *44*, 116773.
[13] Zanini et al., *Adv Funct Materials* **2024**, *34*, 2406916.
[14] Luitz et al., *Adv Eng Mater* **2023**, *25*, 2201927.
[15] Zan et al., *J. Phys. D: Appl. Phys.* **2022**, *55*, 444004.
[16] Du Plessis et al., *3D Print. Addit. Manuf.* **2018**, 5, 227.
[17] Choren et al. *J. Mater. Sci.* **2013**, 48, 5103
